# Regiocontrolled Microwave Assisted Bifunctionalization of 7,8-Dihalogenated Imidazo[1,2-*a*]pyridines: A One Pot Double-Coupling Approach

**DOI:** 10.3390/molecules170910683

**Published:** 2012-09-06

**Authors:** Emilie Marie, Sébastien Bouclé, Cécile Enguehard-Gueiffier, Alain Gueiffier

**Affiliations:** Recherche et Innovation en Chimie Médicinale, UMR INRA 1282 Infectiologie et Santé Publique, Université F. Rabelais, 31 avenue Monge, F-37200 Tours, France; Email: emilie.marie-2@etu.univ-tours.fr (E.M.); s.boucle@hotmail.fr (S.B.); alain.gueiffier@univ-tours.fr (A.G.)

**Keywords:** imidazo[1,2-*a*]pyridine, metallo-catalyzed cross-coupling reaction, microwave irradiation, Suzuki-Miyaura cross-coupling, Sonogashira cross-coupling, cyanation, Buchwald-Hartwig cross-coupling

## Abstract

The reactivity of the 7-chloro-8-iodo- and 8-chloro-7-iodoimidazo[1,2-*a*]pyridines **1a**–**e** diversely substituted on the 2 position, towards Suzuki-Miyaura, Sonogashira, and Buchwald-Hartwig cross-coupling reactions as well as cyanation was evaluated. Various methodologies are proposed to introduce aryl, heteroaryl, alkyne, amine or cyano groups in the two positions depending on the nature of the substituent present in position 2. In both series, the substitution of the iodine atom was totally regioselective and the difficulty was to substitute the chlorine atom in a second step. Until now, only hetero(aryl) groups could be introduced though Suzuki-Miyaura cross-coupling. We overcame this problem evaluating both regioisomers in parallel. The double coupling approach was also studied allowing the one pot Suzuki/Suzuki, cyanation/Sonogashira and cyanation/Buchwald reactions leading to polyfunctionnalized imidazo[1,2-*a*]pyridines.

## 1. Introduction

Over the last decades, the imidazo[1,2-*a*]pyridine ring has been considered an important scaffold for biomolecules in medicinal chemistry, with a broad spectrum of potential therapeutic properties such as, for example, antibacterial [1], HIV inhibitory [2], anti-inflammatory [3] and anticancer [4] properties. Moreover, several drug formulations containing imidazo[1,2-*a*]pyridine derivatives, are marketed in the anxiolytic/hypnotic area (zolpidem, alpidem) and GDR/peptic ulcer therapy (zolimidine). Therefore, convergent, rapid, and easy to implement functionalization routes are still needed to introduce various modulations on this skeleton, in order to complete the putative biological activities evaluation of these series of compounds.

In the course of our work evaluating the chemical and pharmacological properties of the imidazo[1,2-*a*]pyridine series, we further pursued investigations on methods of functionalization which would allow the rapid preparation of a number of structural variants. We thus became interested in the regiocontrolled substitution of 7,8-dihalogenated imidazo[1,2-*a*]pyridine derivatives. The introduction of various substituents such as aryl, heteroaryl, cyano and amino groups was studied and our results in this area are the subject of this manuscript. Analogous works on double-coupling approach in positions 3 and 6, or positions 6 and 8 of this scaffold were already presented by other groups [5,6].

Recently in the literature, a few pharmacomodulation studies of the imidazo[1,2-*a*]pyridine scaffold required this type of 7,8-disubstitution pattern for their purposes. Kettle *et al.* described a two-step introduction of aryl groups in these 7 and 8 positions [7]. In our work, we propose an alternative one-step procedure. Moreover, Trabanco *et al.* synthesized 7-aryl-8-cyano or 7-aryl-8-chloroimidazo[1,2-*a*]pyridine derivatives starting from the convenient 7,8-dihalogenated starting material [8]. Concerning older publications [9,10,11], authors usually introduced the cyano group on the pyridine ring before cyclisation, increasing the number of synthetic steps. 

## 2. Results and Discussion

In order to control the regioselectivity of the disubstitution pattern in positions 7 and 8 of the imidazo[1,2-*a*]pyridine scaffold, we chosen as starting materials the 7-chloro-8-iodo- and 8-chloro-7-iodoimidazo[1,2-*a*]pyridines **1a**–**e** diversely substituted on the 2 position. Both series were studied concomitantly in order to offer the largest scope of functionalization. To our knowledge, the analogous 7-bromo-8-iodo- and 8-bromo-7-iodoimidazo[1,2-*a*]pyridines are still not described in the literature.

Compounds **1a**–**e** were obtained in 71–100% yields by condensation of 2-amino-3-chloro-4-iodopyridine or 2-amino-4-chloro-3-iodopyridine, with the corresponding α-halogenoketones. Different groups (phenyl, ethyl carboxylate, methyl) were considered in position 2 to overview the influence of this position on the reactivity of the 7 and 8 positions of the imidazo[1,2-*a*]pyridine series towards various cross-coupling reactions. Indeed, such an influence of the 2-substituent on the reactivity of not only position 3 [12], but also of position 6 [13], was previously noticed by our group.

### 2.1. Regioselective Substitution of the Iodine Atom of Compounds **1a**–**e**

**Scheme 1:**
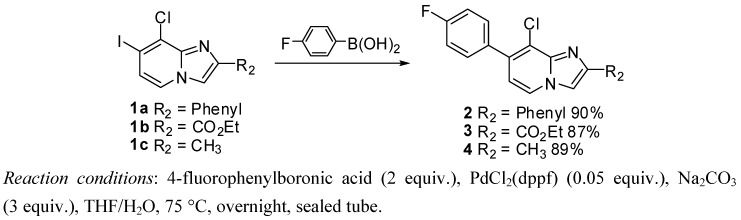
Suzuki cross-coupling reaction on position 7 of compounds **1a**–**c** (isolated yields).

**Scheme 2:**
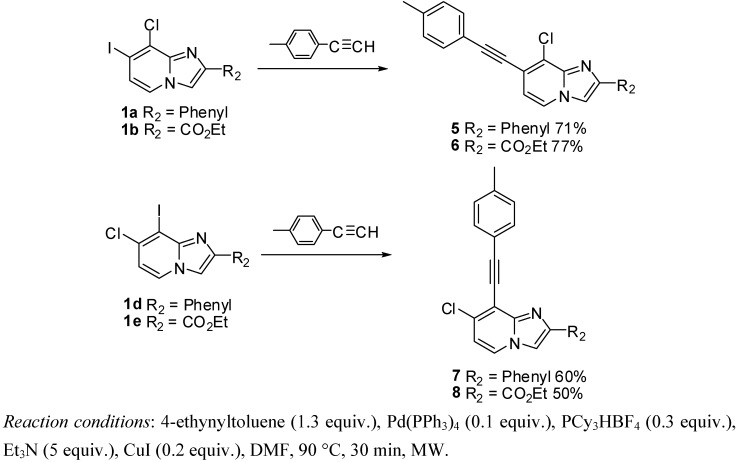
Sonogashira cross-coupling reaction on compounds **1a**, **1b**, **1d**, **1e** (isolated yields).

**Scheme 3:**
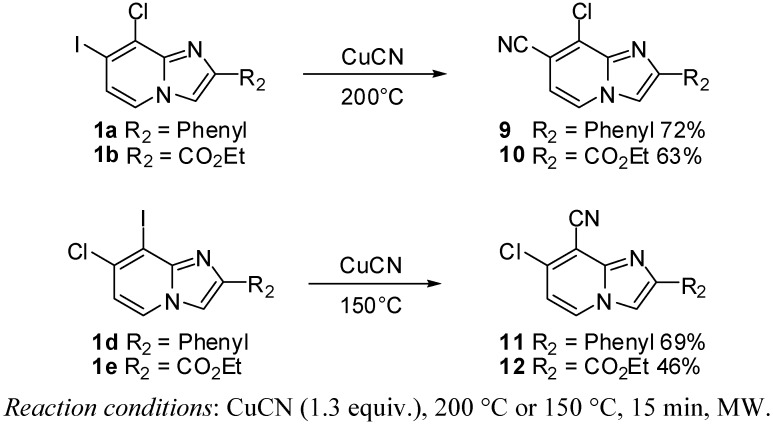
Cyanation on compounds **1a**, **1b**, **1d**, **1e** (isolated yields).

**Scheme 4:**
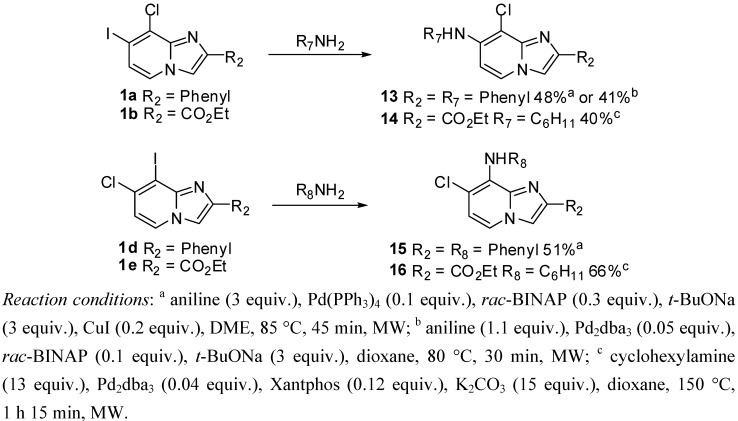
Buchwald-Hartwig cross-coupling on compounds **1a**, **1b**, **1d**, **1e** (isolated yields).

Initial work focused on the regioselective substitution of iodine atom of compounds **1a**–**e**. Three classical cross-coupling reactions were studied: Suzuki-Miyaura, Sonogashira and Buchwald-Hartwig reactions, as well as cyanation reactions (Scheme 1, Scheme 2, Scheme 3 and Scheme 4). 

#### 2.1.1. Suzuki Cross-Coupling on Compounds **1a**–**c**

Suzuki cross-coupling standard conditions were first applied to **1a**–**c** using 4-fluorophenylboronic acid (2 equiv.), PdCl_2_(dppf) (0.05 equiv.), Na_2_CO_3_ (3 equiv.) in a mixture of THF-H_2_O at 75 °C overnight leading to compounds **2**–**4** in 87%–90% yields (Scheme 1). Total conversion and regioselectivity were observed. No influence of the 2 position was noticed.

#### 2.1.2. Sonogashira Cross-Coupling on Compounds **1a**–**e**

Optimization of Sonogashira cross-coupling conditions was conducted in position 8 of compound **1d** using 4-ethynyltoluene (3 equiv.), PdCl_2_(dppf) (0.05 equiv.), TEA (5 equiv.) and CuI (0.1 equiv.) in DMF at 90 °C for 1 h under microwave irradiation [14]. A partial conversion was observed (NMR yield of 29%) and a large amount of starting material was recovered, that could not be separated from the attempted product. A methodology developed in our laboratory using 4-ethynyltoluene (1.3 equiv.), Pd(PPh_3_)_4_ (0.1 equiv.), PCy_3_HBF_4_ (0.3 equiv.) in the presence of *t*-BuONa (3 equiv.) and CuI (0.4 equiv.) in DMF at 100 °C for 20 min under microwave irradiation was then tested. A total dehalogenation occurred in position 8. Thus, we decided to work at lower temperature (90 °C), to change the base to Et_3_N (5 equiv.) and to lower the amount of CuI (0.2 equiv.). Under these conditions, a total conversion was observed allowing the purification of the attempted compounds **7** and **8** in moderate yields (60 and 50%, respectively) (Scheme 2). The position 7 appeared to be more reactive towards these Sonogashira conditions than position 8, leading to compounds **5** and **6** in 71% and 77% yields respectively (Scheme 2). The presence of N_1_ in close vicinity of position 8 may explain its lower reactivity. Moreover, the 2-ester function seems to lower the reactivity of the 8 position towards the Sonogashira cross-coupling.

#### 2.1.3. Cyanation on Compounds **1a**–**e**

Then, we considered the cyanation of compounds **1** (Scheme 3). According to a literature procedure, reaction of **1a** with CuCN (1.6 equiv.) in acetonitrile at 160 °C for 30 min under microwaves irradiation led to **9** in 32% yield [8]. We then changed the solvent to DMF and reaction of **1a** or **1b** with CuCN (1.3 equiv.) at 200 °C for 15 min under microwave irradiation, afforded **9** and **10** in respective yields of 72 and 63%. Applying the same conditions to compounds **1d**–**e** led to formation of dehalogenated compounds. The 8-cyano compounds **11** and **12** were obtained after reaction at 150 °C for 15 min in 69% and 46% yields, respectively. Again, the 2-ester function seems to lower the reactivity of the 8 position towards the cyanation reaction.

#### 2.1.4. Buchwald-Hartwig Cross-Coupling on Compounds **1a**–**e**

Finally, we carried out a short study of Buchwald-Hartwig amination to optimize the aniline cross-coupling conditions, depending on the substituent present in position 2 of the imidazo[1,2-*a*]pyridine ring (Scheme 4). A nucleophilic substitution was first attempted following the procedure described by Tresadern *et al.* for the coupling of alkylpiperazine to 7-iodo-8-cyanoimidazo[1,2-*a*]pyridine derivatives [9]. Treatment of **1a** with *N*-methylpiperazine (3 equiv.), DIPEA (4 equiv.) in acetonitrile at 180 °C for 1 h under microwave irradiation led exclusively to a mixture of starting material and deiodinated compound. The 8-cyano group appears to greatly increase the reactivity of the adjacent position, allowing a nucleophilic substitution. 

In view to study one-pot heterogeneous double-coupling of aryl and amine groups in positions 7 and 8, Pd(PPh_3_)_4_ was preferred as catalyst in a first attempt. Thus, the reaction was achieved using aniline (1 equiv.), Pd(PPh_3_)_4_ (0.1 equiv.), Xantphos (0.3 equiv.), K_2_CO_3_ (10 equiv.) in dioxane, but only starting material was recovered after 90 min at 100 °C under microwave irradiation. In the same way, only traces of the attempted compounds **13** were obtained using the following conditions: aniline (3 equiv.), Pd(PPh_3_)_4_ (0.1 equiv.), *rac*-BINAP (0.3 equiv.), *t*-BuONa (3 equiv.) in DME at 85 °C after 2 h 30 min under microwave irradiation. Therefore, we attempted these last conditions but with a catalytic amount of CuI (0.2 equiv.). We noticed that the reaction was completed after 45 min at 85 °C under microwaves and we obtained **13** in 48% yield. Under the same conditions, we synthesized **15** in 51% yield (Scheme 4).

Several metallo-catalyzed procedures commonly used for aminations were also applied to compound **1a**. Only traces of the attempted compounds **13** were obtained using the following conditions: aniline (1 equiv.), Pd(OAc)_2_ (0.1 equiv.), *rac*-BINAP (0.3 equiv.), *t*-BuONa (3 equiv.) in toluene for 90 min at 90 °C under microwave irradiation. Nevertheless, compound **13** was afforded in 41% yield using aniline (1.1 equiv.), Pd_2_dba_3_ (0.05 equiv.), *rac*-BINAP (0.1 equiv.), *t*-BuONa (3 equiv.) in dioxane at 80 °C for 30 min in a microwave apparatus. Thus, amination in positions 7 and 8 may be achieved through different methodologies with similar yields of around 40%–50%.

The presence of the ester group in compounds **1b** and **1e** prevented us from using *t*-BuONa as base for the amination reaction. The different approaches evaluated to introduce the aniline were unsuccessful. We then decided to work with cyclohexylamine. The coupling reaction was first conducted on **1b** using cyclohexylamine (2 equiv.), Pd(PPh_3_)_4_ (0.1 equiv.), Xantphos (0.2 equiv.), K_2_CO_3_ (15 equiv.) in DMF at 160 °C during 1 h under microwave irradiation. This reaction did not afford the expected product **14**. The reaction was then attempted on **1e** using cyclohexylamine (13 equiv.), Pd_2_dba_3_ (0.04 equiv.), Xantphos (0.12 equiv.), K_2_CO_3_ (15 equiv.) in dioxane for 1 h 15 min at 150 °C under microwaves, leading to **16** in 66% yield (Table 2). These last conditions applied to **1b** led to **14** in 40% yield (Scheme 4). 

### 2.2. Substitution of the Chlorine Atom in Position 8 of Compounds **2**–**6**, **9**–**10**, **14**

In a second part, we focused on the substitution of the chlorine atom in position 8 of the 7-substituted imidazo[1,2-*a*]pyridines **2**–**6**, **9**–**10**, **14**. The reactivity of this position was evaluated towards the Suzuki-Miyaura cross-coupling reaction (Table 1). In a first attempt, the reaction was performed on the ester compound **3** using 4-tolylboronic acid (1.8 equiv.), Pd(PPh_3_)_4_ (0.05 equiv.), Na_2_CO_3_ (1.5 equiv.), in DME/H_2_O at 75 °C in a sealed tube, leading to the target compound **17** in 53% yield. Nevertheless, a reaction time of 22 h was required, and addition of amounts of 4-tolylboronic acid to the reaction mixture until 2.5 equivalents of 4-tolylboronic acid were used. A higher temperature was incompatible with the ester group. These conditions were greatly improved using 4-tolylboronic acid (1.4 equiv.), Pd(PPh_3_)_4_ (0.1 equiv.), K_2_CO_3_ (2 equiv.) in dioxane/EtOH for 15 min at 150 °C under microwave irradiation, leading to **17** in 95% yield. In the same conditions, **18** was obtained in 76% yield. Starting from the 2-phenyl or 2-methyl compounds **2** or **4**, compounds **19**–**20** and **21**–**22** were obtained using classical conditions (boronic acid (1.4 or 2 equiv.), Pd(PPh_3_)_4_ (0.05 equiv.), Na_2_CO_3_ (2 equiv.), DME/H_2_O at 120 °C under microwave irradiation) in good yields (80 to 90%). In these conditions, no influence of the nature of the 2-substituent was noticed on the reactivity of the 8 position.

**Table 1:** Functionalization in position 8 of compounds **2**–**4**. 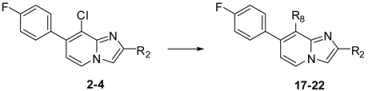

S.M.	Product	Isolated Yield (%)	S.M.	Product	Isolated Yield (%)
**3**	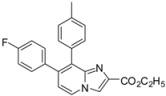	95 ^a^	**3**	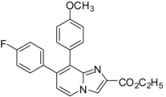	76 ^a^
	**17**			**18**	
**2**	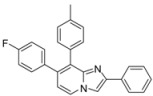	90 ^b^	**4**	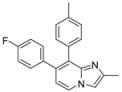	84 ^b^
	**19**			**20**	
**2**	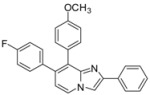	82 ^b^	**4**	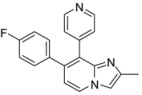	80 ^b^
	**21**			**22**	

*Reaction conditions*: ^a^ RB(OH)_2_ (1.4 equiv.), K_2_CO_3_ (2 equiv.), Pd(PPh_3_)_4_ (0.1 equiv.), dioxane/EtOH, 150 °C, MW or ^b^ RB(OH)_2_ (1.4 or 2 equiv.), Na_2_CO_3_ (2 equiv.), Pd(PPh_3_)_4_ (0.05 equiv.), DME/H_2_O, 120 °C, MW.

The Suzuki coupling reaction was then extended to the diversely 7-substituted imidazo[1,2-*a*]pyridines **5**–**6**, **9**–**10**, **14** (Table 2). As previously described, two synthetic routes were applied depending on the 2-substituent. Reaction of **5** or **9** with various (hetero)arylboronic acids (1.4 or 2 equiv.) in the presence of Pd(PPh_3_)_4_ (0.05 equiv.), Na_2_CO_3_ (2 equiv.) in DME/H_2_O at 120 °C under microwaves irradiation gave **23** to **25** in moderate to good yields (19%–71%). Reaction of the ester compounds **6**, **10**, **14** with (hetero)arylboronic acids (1.4 or 2 equiv.) in the presence of Pd(PPh_3_)_4_ (0.1 equiv.), K_2_CO_3_ (2 equiv.) in dioxane/EtOH at 150 °C under microwave irradiation led to 27 to 35% of compounds **26**–**30**. 

**Table 2:** Functionalization in position 8 of compounds **5**–**6**, **9**–**10**, **14**. 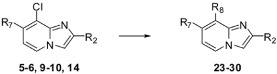

S.M.	Products	Isolated Yield (%)	S.M.	Products	Isolated Yield (%)
**5**	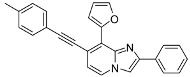	19 ^a^	**9**	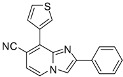	71 ^a^
	**23**			**24**	
**9**	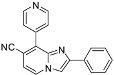	42 ^a^	**14**	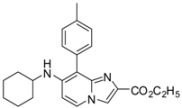	27 ^b^
	**25**			**26**	
**6**	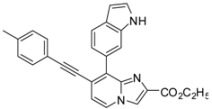	35 ^b^	**10**	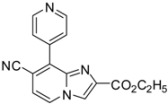	31 ^b^
	**27**			**28**	
**10**	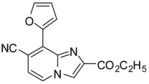	34 ^b^	**14**	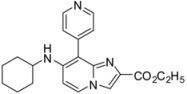	28 ^b^
	**29**			**30**	

*Reaction conditions*: ^a^ RB(OH)_2_ (1.4 or 2 equiv.), Na_2_CO_3_ (2 equiv.), Pd(PPh_3_)_4_ (0.05 equiv.), DME/H_2_O, 120 °C, MW; ^b^ RB(OH)_2_ (1.4 or 2 equiv.), K_2_CO_3_ (2 equiv.), Pd(PPh_3_)_4_ (0.1 equiv.), dioxane/EtOH, 150 °C, MW.

Due to the lower reactivity of the chlorine atom in position 8 of the imidazo[1,2-*a*]pyridines **2**–**6**, **9**–**10**, **14**, only 8-(hetero)aryl-7-substituted compounds could be obtained through Suzuki cross-coupling reactions. No cyanation, Sonogashira or amination reaction could be achieved at this position. Nevertheless, the problem of chlorine reactivity could be solved starting from the regioisomers **7**–**8**, **11**, **15** iodinated in position 8. 

### 2.3. Substitution of the Chlorine Atom in Position 7 of Compounds **7**–**8**, **11**, **15**

Using the previously established experimental conditions, four examples of 7-arylimidazo[1,2-*a*]pyridines **31**–**34** were obtained presenting an alkyne, cyano or amino group in position 8 (44 to 69% yields) (Table 3). For compound **34**, we noticed the formation of a dehalogenated compound during the Suzuki cross-coupling reaction, and a higher amount of boronic acid was then required to complete the reaction. 

**Table 3:** Functionalization in position 7 of compounds **7**–**8**, **11**, **15**. 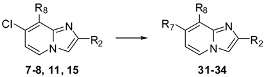

S.M.	Products	Isolated Yield (%)	S.M.	Products	Isolated Yield (%)
**7**	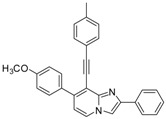	56 ^a^	**8**	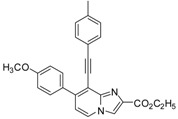	69 ^b^
	**31**			**32**	
**11**	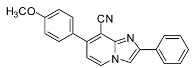	61 ^a^	**15**	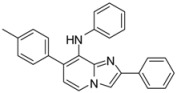	44 ^a^
	**33**			**34**	

*Reaction conditions*: ^a^ RB(OH)_2_ (1.4 or 2 or 5 equiv.), Na_2_CO_3_ (2 equiv.), Pd(PPh_3_)_4_ (0.05 equiv.), DME/H_2_O, 120 °C, MW; ^b^ RB(OH)_2_ (1.4 equiv.), K_2_CO_3_ (2 equiv.), Pd(PPh_3_)_4_ (0.1 equiv.), dioxane/EtOH, 150 °C, MW.

### 2.4. Double Coupling Approach

Finally, the 7,8-diarylation of the imidazo[1,2-*a*]pyridine scaffold in a one-pot approach was evaluated (Table 4). Starting from compound **1a**, optimization of the Suzuki double coupling led to the following conditions: first boronic acid (1 equiv.), Na_2_CO_3_ (3 equiv.), Pd(PPh_3_)_4_ (0.05 equiv.) in a mixture DME/H_2_O for 30 min at 95 °C under microwave irradiation, then second boronic acid (1.4 equiv.), Pd(PPh_3_)_4_ (0.05 equiv.) for 30 min at 120 °C under microwave irradiation. Compounds **18**, **35** and **36** were obtained in 43 to 72% yields. Starting from the regioisomer **1d**, 2 equivalents of both boronic acids were required.

One pot cyanation/Sonogashira and cyanation/Buchwald double couplings could be performed starting from compounds **1d**–**e** leading to compounds **38**–**41** in 32 to 67% yields. After optimization, the one pot cyanation/Sonogashira conditions required CuCN (1.2 equiv.) in DMF at 90 °C for 4 h under microwave irradiation, then 4-ethynyltoluene (1.3 equiv.), Et_3_N (5 equiv.), Pd(PPh_3_)_4_ (0.1 equiv.), PCy_3_HBF_4_ (0.3 equiv.), CuI (0.2 equiv.) at 130 °C for 1 h under microwave irradiation. The one pot cyanation/Buchwald double coupling was performed using CuCN (1.2 equiv.) in DMF at 90 °C for 4 h under microwave irradiation, then DIPEA (2 equiv.), 4-fluorophenylpiperidine (2 equiv.) at 130 °C for 1 h under microwave irradiation. These results encouraged us to study other heterogeneous double couplings.

**Table 4:** One pot double-coupling approach applied to compounds **1a**, **1d** and **1e**.

S.M.	Products	Isolated Yield (%)	S.M.	Products	Isolated Yield (%)
**1a**	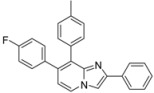	72 ^a^	**1a**	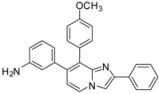	44 ^a^
	**18**			**35**	
**1a**	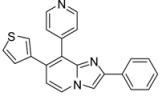	43 ^a^	**1d**	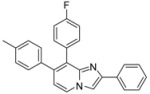	47 ^b^
	**36**			**37**	
**1d**	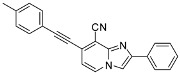	32 ^c^	**1d**	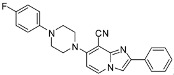	67 ^d^
	**38**			**39**	
**1e**	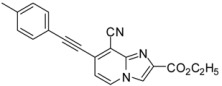	43 ^c^	**1e**	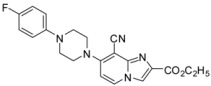	54 ^d^
	**40**			**41**	

*Reaction conditions*: ^a^ R_1_B(OH)_2_ (1 equiv.), Na_2_CO_3_ (3 equiv.), Pd(PPh_3_)_4_ (0.05 equiv.), DME/H_2_O, 95 °C, 30 min, MW then R_2_B(OH)_2_ (1.4 equiv.), Pd(PPh_3_)_4_ (0.05 equiv.), 120 °C, 30 min, MW; ^b^ R_1_B(OH)_2_ (2 equiv.), Na_2_CO_3_ (3 equiv.), Pd(PPh_3_)_4_ (0.05 equiv.), DME/H_2_O, 95 °C, 1 h 30 min, MW then R_2_B(OH)_2_ (2 equiv.), Pd(PPh_3_)_4_ (0.05 equiv.), 120 °C, 30 min, MW; ^c^ CuCN (1.2 equiv.), DMF, 90 °C, 4 h, MW then 4-ethynyltoluene (1.3 equiv.), Et_3_N (5 equiv.), Pd(PPh_3_)_4_ (0.1 equiv.), PCy_3_HBF_4_ (0.3 equiv.), CuI (0.2 equiv.), 130 °C, 1 h, MW; ^d^ CuCN (1.2 equiv.), DMF, 90 °C, 4 h, MW then DIPEA (2 equiv.), 4-fluorophenylpiperidine (2 equiv.), 130 °C, 1 h, MW.

## 3. Experimental

### 3.1. General

Commercial reagents were used as received without additional purification. The microwave experiments were performed with a CEM Discover SP® equipped with an Explorer hybrid unit, only the set point temperature was adjusted (set point pressure: 17 bar, set point power: 200 W). The compounds were purified on a Flashsmart® flash chromatography system and columns from AIT® and Macherey-Nagel® were used. ^1^H and ^13^C-NMR spectra were recorded on a Brüker 300 MHz spectrometer in CDCl_3_ or in DMSO-*d_6_*. The possible inversion of two values in the NMR spectra is expressed by an asterisk. The melting points were determined in a capillary apparatus Stuart® SMP3 and are uncorrected. 

### 3.2. Synthesis

#### 3.2.1. Obtention of the Aminochloroiodopyridines

*2,3-Dichloro-4-iodopyridine* [8,15]. To a solution of *n*-butyllithium (27.6 mL, 69 mmol, 2.5 M in hexanes) in dry Et_2_O (153 mL) cooled at −78 °C, was added dropwise 2,2,6,6-tetramethylpiperidine (11.64 mL, 69 mmol). The mixture was stirred at −78 °C for 10 min and a solution of 2,3-dichloropyridine (10 g, 67.57 mmol) in dry THF (75 mL) was added dropwise. The resulting mixture was stirred at −78 °C for 30 min and a solution of I_2_ (25.38 g, 100 mmol) in dry THF (75 mL) was added. The reaction was cooled to room temperature overnight, quenched with a saturated aqueous solution of Na_2_S_2_O_3_ and extracted with EtOAc (3 × 50 mL). The combined organic layer was washed with a saturated aqueous solution of NaHCO_3_, dried with anhydrous MgSO_4_ and evaporated *in vacuo*. The residue was purified on column chromatography [silica gel, cyclohexane/EtOAc (7:3)] to give a pale cream solid. M.p. = 110 °C (Ref. [8] 113 °C). 67% yield. ^1^H-NMR (CDCl_3_) δ 7.89 (d, 1H, *J* = 5.1 Hz, H-6), 7.73 (d, 1H, *J* = 5.1 Hz, H-5). ^13^C-NMR (CDCl_3_) δ 148.5, 146.6, 135.4, 134.0, 111.2.

*2-Amino-3-chloro-4-iodopyridine* [8]. A mixture of 2,3-dichloro-4-iodopyridine (4 g, 14.71 mmol) in NH_4_OH 28% (75 mL) was heated in a Parr bomb at 129 °C for 16 h. The reaction was cooled to room temperature and CH_2_Cl_2_ (100 mL) was added. The mixture was extracted with CH_2_Cl_2_ (3 × 100 mL), dried with anhydrous MgSO_4_ and evaporated *in vacuo*. The residue was purified by flash chromatography [silica gel, cyclohexane/EtOAc (7:3)] and a white solid was obtained. M.p. = 110 °C. 38% yield. ^1^H-NMR (CDCl_3_) δ 7.54 (d, 1H, *J* = 5.3 Hz, H-6), 7.08 (d, 1H, *J* = 5.3 Hz, H-5), 5.32 (s, 2H, NH_2_). ^13^C-NMR (CDCl_3_) δ 154.6, 145.6, 124.7, 119.4, 109.8.

*t-Butyl-(4-chloropyridin-2-yl)carbamate* [16,17]. To a solution of 2-amino-4-chloropyridine (3 g, 23.34 mmol) in *t*-BuOH (43 mL), di-*t-*butyl dicarbonate (5.6 g, 25.67 mmol) was added. The mixture was stirred at 30 °C overnight. A solid appeared and was filtered off, washed with *n*-hexane and diethyl ether to give a white solid. M.p. = 150 °C. 77% yield. ^1^H-NMR (DMSO-*d*_6_) δ 10.14 (bs, 1H, NH), 8.22 (d, 1H, *J* = 5.4 Hz, H-6), 7.88 (d, 1H, *J* = 1.9 Hz, H-3), 7.14 (dd, 1H, *J* = 5.4–1.9 Hz, H-5), 1.47 (s, 9H, CH_3_). ^13^C-NMR (DMSO-*d*_6_) δ 153.69, 152.66, 149.34, 143.84, 118.29, 111.65, 80.15, 27.95.

*t-Butyl-(4-chloro-3-iodopyridin-2-yl)carbamate* [17]. To a solution of *t*-butyl-(4-chloropyridin-2-yl)carbamate (9 g, 39.5 mmol) in anhydrous THF (250 mL), TMEDA (14.4 mL) was added under nitrogen atmosphere and cooled to −70 °C. To the mixture, 2.5 M in hexane *n*-BuLi (39.6 mL, 98 mmol) was added dropwise over a period of 30 min. The mixture was stirred at −70 °C for 1 h and then treated dropwise with a solution of I_2_ (50 g, 198 mmol) in anhydrous THF (30 mL) at −70 °C. After the addition was complete, the reaction was stirred at −70 °C for 30 min and then allowed to warm to room temperature. The mixture was treated with an aqueous saturated solution of Na_2_S_2_O_3_ and stirred for 30 min. The solution was extracted with EtOAc (3 × 200 mL). The combined organic layer was washed with brine, dried with anhydrous MgSO_4_ and evaporated under reduce pressure. The product was purified by flash chromatography [silica, cyclohexane/EtOAc (7:3)] to give a white solid. M.p. = 183 °C. 75% yield. ^1^H-NMR (DMSO-*d*_6_) δ 9.48 (s, 1H, NH), 8.30 (d, 1H, *J =* 5.1 Hz, H-6), 7.47 (d, 1H, *J* = 5.1 Hz, H-5), 1.45 (s, 9H, CH_3_). ^13^C-NMR (DMSO-*d*_6_) δ 154.9, 152.6, 149.0, 148.4, 122.0, 99.2, 79.4, 28.1.

*2-Amino-4-chloro-3-iodopyridine* [7,8,17]. A suspension of *t*-butyl-(4-chloro-3-iodopyridin-2-yl)carbamate (6 g, 23.5 mmol) in 48% hydrobromic acid (12 mL) was heated at 100 °C for 10 min to give a clear solution. The mixture was cooled, treated with ice and made basic with 10 M NaOH solution. The precipitated product was filtered off, washed with H_2_O to give a cream solid. M.p. = 111 °C. 97% yield. ^1^H-NMR (DMSO-*d*_6_) δ 7.84 (d, 1H, *J* = 5.2 Hz, H-6), 6.72 (d, 1H, *J* = 5.2 Hz, H-5), 6.44 (bs, 2H, NH_2_). ^13^C-NMR (DMSO-*d*_6_) δ 161.0, 148.4, 147.7, 113.0, 81.4.

#### 3.2.2. General Procedure for Cyclization

*8-Chloro-7-iodo-2-phenylimidazo[1,2-a]pyridine* (**1a**). Method A. To a solution of 2-amino-3-chloro-4-iodopyridine (1 g, 3.94 mmol) in EtOH (10 mL), was added bromoacetophenone (1.56 g, 7.87 mmol). The mixture was stirred at 65 °C overnight. The solution was then evaporated to dryness *in vacuo*. The residue was dissolved in CH_2_Cl_2_ (50 mL) and the resulting solution made basic by the addition of a saturated aqueous solution of Na_2_CO_3_. The solution was extracted with CH_2_Cl_2_ (3 × 100 mL), the combined organic layers were dried with anhydrous MgSO_4_ and evaporated under reduced pressure. The crude product was purified by flash chromatography [silica gel, cyclohexane/EtOAc (7:3)]. M.p. = 101 °C. 97% yield. ^1^H-NMR (CDCl_3_) δ 7.96 (dd, 2H, *J* = 8.2–1.3 Hz, Ph-2,6), 7.88 (s, 1H, H-3), 7.80 (d, 1H, *J* = 7.0 Hz, H-5), 7.44 (m, 2H, Ph-3,5), 7.35 (m, 1H, Ph-4), 7.12 (d, 1H, *J =* 7.0 Hz, H-6). ^13^C-NMR (CDCl_3_) δ 146.8, 143.0, 132.8, 128.7, 128.5, 126.4, 123.9, 121.7, 110.0, 92.1(1C not found). 

*Ethyl 8-chloro-7-iodoimidazo[1,2-a]pyridine-2-carboxylate* (**1b**). Method B. To a solution of 2-amino-3-chloro-4-iodopyridine (1 g, 3,94 mmol) in DME (18 mL), was added ethyl bromopyruvate (1.16 g, 5.91 mmol). The mixture was stirred at room temperature overnight. The solution was evaporated *in vacuo* to dryness and EtOH (18 mL) was added. The mixture was stirred at 78 °C for 2 h. After cooling to room temperature, the solution was evaporated to dryness *under vacuum*. The residue was dissolved in CH_2_Cl_2_ (50 mL) and the resulting solution made basic by the addition of a saturated solution of Na_2_CO_3_. The solution was extracted with CH_2_Cl_2_ (3 × 100 mL), the combined organic layers were dried with anhydrous MgSO_4_ and evaporated under reduced pressure. The crude product was washed with diethyl ether and *n*-hexane to give a pale cream solid. M.p. = 249 °C. 95% yield. ^1^H-NMR (CDCl_3_) δ 8.66 (s, 1H, H-3), 8.33 (d, 1H, *J* = 6.9 Hz, H-5), 7.41 (d, 1H, *J* = 6.9 Hz, H-6), 4.32 (q, 2H, *J* = 7.2 Hz, CH_2_), 1.32 (t, 3H, *J* = 7.2 Hz, CH_3_). ^13^C-NMR (CDCl_3_) δ 162.6, 142.3, 136.1, 127.1, 123.0, 120.8, 97.4, 61.0, 14.7 (1C not found). 

*8-Chloro-7-iodo-2-methylimidazo[1,2-a]pyridine* (**1c**). Method A. 2-Amino-3-chloro-4-iodopyridine (1 g, 3.94 mmol) and chloroacetone (1.46 g, 15.75 mmol) were used. M.p. = 136 °C. Quantitative yield. ^1^H-NMR (CDCl_3_) δ 7.72 (d, 1H, *J* = 6.9 Hz, H-5), 7.38 (s, 1H, H-3), 7.07 (d, 1H, *J* = 6.9 Hz, H-6), 2.48 (s, 3H, CH_3_). ^13^C-NMR (CDCl_3_) δ 144.6, 131.9, 127.5, 123.6, 121.1, 111.6, 91.5, 14.4. 

*7-Chloro-8-iodo-2-phenylimidazo[1,2-a]pyridine* (**1d**). Method A. 2-Amino-4-chloro-3-iodopyridine (1 g, 3.94 mmol) and bromoacetophenone (1.56 g, 7.87 mmol) were used. M.p. = 197 °C. 72% yield. ^1^H-NMR (CDCl_3_) δ 8.03–7.94 (m, 4H, Ph-2,6, H-3, H-5), 7.44 (m, 2H, Ph-3,5), 7.36 (m, 1H, Ph-4), 6.84 (d, 1H, *J* = 7.0 Hz, H-6). ^13^C-NMR (DMSO-*d_6_*) δ 145.5, 145.2, 135.1, 133.1, 128.8, 128.1, 127.2, 125.7, 113.1, 111.5, 88.6. 

*Ethyl 7-chloro-8-iodoimidazo[1,2-a]pyridine-2-carboxylate* (**1e**). Method B. 2-Amino-4-chloro-3-iodopyridine (1 g, 3.94 mmol) was used as starting material. M.p. = 208 °C. 71% yield. ^1^H-NMR (DMSO-*d_6_*) δ 8.71 (s, 1H, H-3), 8.55 (d, 1H, *J* = 7.3 Hz, H-5), 7.16 (d, 1H, *J* = 7.3 Hz, H-6), 4.33 (q, 2H, *J* = 7.0 Hz, CH_2_), 1.32 (t, 3H, *J* = 7.0 Hz, CH_3_). ^13^C-NMR (DMSO-*d_6_*) δ 162.2, 145.4, 137.0, 136.2, 127.9, 120.4, 114.5, 89.9, 60.5, 14.3. 

#### 3.2.3. Suzuki Cross-Coupling on Compounds **1a**–**c**

*8-Chloro-7-(4-fluorophenyl)-2-phenylimidazo[1,2-a]pyridine* (**2**). Method C. To a solution of **1a** (500 mg, 1.41 mmol) in a mixture of THF (2.7 mL) and water (0.3 mL) in a sealed tube, were added 4-fluorophenylboronic acid (296 mg, 2.11 mmol), Na_2_CO_3_ (449 mg, 4.24 mmol) and PdCl_2_(dppf) (58 mg, 0.07 mmol, 5 mol%). The mixture was stirred at 75 °C for 1 h 30 min, 4-fluorophenylboronic acid (99 mg, 0.5 equiv.) and a few amount of PdCl_2_(dppf) were added. After overnight stirring at 75 °C and cooling to room temperature, CH_2_Cl_2_ (50 mL) and water (50 mL) were added and the solution was extracted with CH_2_Cl_2_ (3 × 50 mL). The combined organic layer was dried with anhydrous MgSO_4_ and evaporated under reduced pressure. The crude residues were purified by column chromatography (alumina, CH_2_Cl_2_). M.p. = 211 °C. 90% yield. ^1^H-NMR (CDCl_3_) δ 8.09 (d, 1H, *J* = 6.9 Hz, H-5), 8.02 (m, 2H, Ph-2,6), 7.92 (s, 1H, H-3), 7.52 (dd, 2H, J = 8.8–5.2 Hz, F-Ph-2,6), 7.45 (m, 2H, Ph-3,5), 7.37 (m, 1H, Ph-4), 7.18 (t, 2H, *J* = 8,8 Hz, F-Ph-3,5), 6.80 (d, 1H, *J* = 6.9 Hz, H-6). ^13^C-NMR (CDCl_3_) δ 162.6, 147.1, 134.6, 133.2, 133.1, 131.1, 128.7, 128.3, 126.4, 123.4, 115.5, 114.8, 109.4. (2C not found) Anal. Calcd for C_19_H_12_ClFN_2_: C, 70.70; H, 3.75; N, 8.68. Found: C, 70.94; H, 3.67; N, 8.73. 

*Ethyl 8-chloro-7-(4-fluorophenyl)imidazo[1,2-a]pyridine-2-carboxylate* (**3**). Method C: **1b** (500 mg, 1.43 mmol), 4-fluorophenylboronic acid (300 mg, 2.14 mmol then 100 mg, 0.71 mmol), Na_2_CO_3_ (454 mg, 4.38 mmol) and PdCl_2_(dppf) (58 mg, 0.07 mmol, 5 mol%) were used. M.p. = 199 °C. 87% yield. ^1^H-NMR (CDCl_3_) δ 8.27 (s, 1H, H-3), 8.15 (d, 1H, *J* = 7.2 Hz, H-5), 7.50 (dd, 2H, *J* = 8.7–5.4 Hz, F-Ph-2,6), 7.17 (t, 2H, *J* = 8.7 Hz, F-Ph-3,5), 6.90 (d, 1H, *J* = 7.2 Hz, H-6), 4.46 (q, 2H, *J* = 7.2 Hz, CH_2_), 1.42 (t, 3H, *J* = 7.2 Hz, CH_3_). ^13^C-NMR (CDCl_3_) δ 162.7, 162.7, 143.1, 137.7, 136.0, 132.6, 131.1, 124.1, 121.5, 118.3, 116.4, 115.5, 61.3, 14.3. Anal. Calcd for C_16_H_12_ClFN_2_O_2_: C, 60.29; H, 3.79; N, 8.79. Found: C, 60.51; H, 3.57; N, 8.81.

*8-Chloro-7-(4-fluorophenyl)-2-methylimidazo[1,2-a]pyridine* (**4**). Method C. **1c** (500 mg, 1.71 mmol), 4-fluorophenylboronic acid (359 mg, 2.56 mmol then 120 mg, 0.85 mmol), Na_2_CO_3_ (545 mg, 5.14 mmol) and PdCl_2_(dppf) (70 mg, 0.09 mmol, 5 mol%) were used. M.p. = 164 °C. 89% Yield. ^1^H-NMR (CDCl_3_) δ 8.06 (d, 1H, *J* = 6.9 Hz, H-5), 7.52 (dd, 2H, *J* = 8.7–5.5 Hz, F-Ph-2,6), 7.46 (s, 1H, H-3), 7.19 (t, 2H, *J* = 8.7 Hz, F-Ph-3,5), 6.81 (d, 1H, *J* = 6.9 Hz, H-6), 2.56 (s, 3H, CH_3_). ^13^C-NMR (CDCl_3_) δ 162.7, 144.0, 133.0, 131.1, 123.3, 115.5, 114.7, 111.1, 14.13. (3C not found) Anal. Calcd for C_14_H_10_ClFN_2_: C, 64.50; H, 3.87; N, 10.75. Found: C, 64.36; H, 3.91; N, 10.83. 

#### 3.2.4. General Procedure for Sonogashira Cross-Coupling Reaction

*8-Chloro-7-(p-tolylethynyl)-2-phenylimidazo[1,2-a]pyridine* (**5**). Method D. To a solution of **1a** (500 mg, 1.41 mmol) in DMF (6 mL), were added Et_3_N ((1 mL, 7.06 mmol), PCy_3_HBF_4_ (156 mg, 0.42 mmol), p-tolylacetylene (213 mg, 1.84 mmol), Pd(PPh_3_)_4_ (163 mg, 0.14 mmol) and CuI (50 mg, 0.26 mmol). The mixture was heated at 90 °C by microwave irradiation for 30 min. After cooling to room temperature, water (50 mL) was added and the solution was extracted with EtOAc (3 × 50 mL). The combined organic layers were dried with anhydrous MgSO_4_ and evaporated under reduced pressure. The crude residues were purified by flash chromatography (silica, cyclohexane/EtOAc (7:3)). M.p. = 176 °C. 71% yield. ^1^H-NMR (CDCl_3_) δ 8.04–7.95 (m, 3H, Ph-2,6, H-5), 7.88 (s, 1H, H-3), 7.55–7.40 (m, 4H, Ph-3,5, CH_3_-Ph-2,6), 7.35 (m, 1H, Ph-4), 7.20 (d, 2H, *J* = 7.9 Hz, CH_3_-Ph-3,5), 6.90 (d, 1H, *J* = 6.8 Hz, H-6), 2.39 (s, 3H, CH_3_). ^13^C-NMR (CDCl_3_) δ 147.4, 139.4, 132.9, 131.7, 129.2, 128.7, 128.6, 128.4, 126.4, 123.2, 119.3, 118.6, 115.0, 110.2, 98.1, 84.6, 21.6 (1C not found). Anal. Calcd for C_22_H_15_ClN_2_: C, 77.08; H, 4.41; N, 8.17. Found: C, 77.24; H, 4.46; N, 8.07.

*Ethyl 8-chloro-7-(p-tolylethynyl)imidazo[1,2-a]pyridine-2-carboxylate* (**6**). Method D. **1b** (500 mg, 1.43 mmol), Et_3_N (1 mL, 7.14 mmol), PCy_3_HBF_4_ (158 mg, 0.43 mmol), p-tolylacetylene (215 mg, 1.86 mmol), Pd(PPh_3_)_4_ (165 mg, 0.14 mmol) and CuI (50 mg, 0.26 mmol) were used. M.p. = 211 °C. 77% yield. ^1^H-NMR (CDCl_3_) δ 8.21 (s, 1H, H-3), 8.04 (d, 1H, *J* = 7.2 Hz, H-5), 7.49 (d, 2H, *J* = 8.1 Hz, CH_3_-Ph-2,6), 7.19 (d, 2H, *J* = 8.1 Hz, CH_3_-Ph-3,5), 6.97 (d, 2H, *J* = 7.2 Hz, H-6), 4.46 (q, 2H, *J* = 7.5 Hz, CH_2_), 2.39 (s, 3H, CH_3_), 1.43 (t, 3H, *J* = 7.5 Hz, CH_3_). ^13^C-NMR (CDCl_3_) δ 162.5, 142.7, 139.8, 137.7, 131.8, 129.3, 126.0, 124.0, 120.6, 119.0, 116.5, 99.5, 84.0, 61.4, 21.6, 14.32 (1C not found). Anal. Calcd for C_19_H_15_ClN_2_O_2_: C, 67.36; H, 4.46; N, 8.27. Found: C, 67.49; H, 4.41; N, 8.38.

*7-Chloro-8-(p-tolylethynyl)-2-phenylimidazo[1,2-a]pyridine* (**7**). Method D. **1d** (250 mg, 0.71 mmol), Et_3_N (0.5 mL, 3.53 mmol), PCy_3_HBF_4_ (78 mg, 0.21 mmol), p-tolylacetylene (107 mg, 0.92 mmol), Pd(PPh_3_)_4_ (81 mg, 0.07 mmol) and CuI (25 mg, 0.13 mmol) were used. M.p. = 118 °C. 60% yield. ^1^H-NMR (CDCl_3_) δ 8.00 (m, 3H, H-5, Ph-2,6), 7.84 (s, 1H, H-3), 7.62 (d, 2H, *J* = 8.0 Hz, CH_3_-Ph-2,6), 7.44 (m, 2H, Ph-3,5), 7.34 (m, 1H, Ph-4), 7.20 (d, 2H, *J* = 8.0 Hz, CH_3_-Ph-3,5), 6.85 (d, 1H, *J* = 7.4 Hz, H-6), 2.39 (s, 3H, CH_3_). ^13^C-NMR (CDCl_3_) δ 146.9, 139.3, 133.1, 132.1, 129.1, 128.9, 128.6, 128.3, 126.3, 126.2, 124.5, 119.6, 115.8, 114.2, 108.9, 81.4, 21.6 (1C not found). Anal. Calcd for C_22_H_15_ClN_2_: C, 77.08; H, 4.41; N, 8.17. Found: C, 77.25; H, 4.43; N, 8.19.

*Ethyl 7-chloro-8-(p-tolylethynyl)imidazo[1,2-a]pyridine-2-carboxylate* (**8**). Method D. **1e** (250 mg, 0.71 mmol), Et_3_N (0.5 mL, 3.53 mmol), PCy_3_HBF_4_ (78 mg, 0.21 mmol), p-tolylacetylene (107 mg, 0.92 mmol), Pd(PPh_3_)_4_ (81 mg, 0.07 mmol) and CuI (25 mg, 0.13 mmol) were added. M.p. = 175 °C. 50% yield. ^1^H-NMR (CDCl_3_) δ 8.20 (s, 1H, H-3), 8.10 (d, 1H, *J* = 7.2 Hz, H-5), 7.55 (d, 2H, *J* = 8.0 Hz, CH_3_-Ph-2,6), 7.17 (d, 2H, *J* = 8.0 Hz, CH_3_-Ph-3,5), 6.95 (d, 1H, *J* = 7.2 Hz, H-6), 4.44 (q, 2H, *J* = 7.1 Hz, CH_2_), 2.37 (s, 3H, CH_3_), 1.42 (t, 3H, *J* = 7.1 Hz, CH_3_). ^13^C-NMR (CDCl_3_) δ 162.7, 144.8, 139.5, 137.6, 135.0, 132.0, 129.0, 125.2, 119.2, 118.0, 115.9, 113.8, 102.8, 80.9, 61.3, 21.6, 14.3. Anal. Calcd for C_19_H_15_ClN_2_O_2_: C, 67.36; H, 4.46; N, 8.27. Found: C, 67.21; H, 4.47; N, 8.46.

#### 3.2.5. General Procedure for Cyanation Reaction

*8-Chloro-2-phenylimidazo[1,2-a]pyridine-7-carbonitrile* (**9**). Method E. To a solution of **1a** (400 mg, 1.13 mmol) in DMF (793 µL), CuCN (132 mg, 1.47 mmol) was added. The tube was evacuated and back filled with nitrogen. Then the reaction mixture was heated at 150 °C or 200 °C by microwave irradiation for 15 min. After cooling to room temperature, an aqueous solution of NH_4_OH 10% (50 mL) and CH_2_Cl_2_ (50 mL) were added and the organic layer was washed with an aqueous solution of NH_4_OH (3 × 50 mL), dried with anhydrous MgSO_4_ and evaporated under reduced pressure. The crude residues were purified on column chromatography (silica gel, CH_2_Cl_2_). The reaction mixture was heated at 200 °C by microwave irradiation. M.p. = 200 °C. 72% yield. ^1^H-NMR (CDCl_3_) δ 8.71 (s, 1H, H-3), 8.67 (d, 1H, *J* = 7.0 Hz, H-5), 8.01 (m, 2H, Ph-2,6), 7.48 (m, 2H, Ph-3,5), 7.39 (m, 1H, Ph-4), 7.30 (d, 1H, *J* = 7.0 Hz, H-6). ^13^C-NMR (CDCl_3_) δ 147.4, 140.7, 132.4, 128.9, 128.8, 127.0, 126.7, 125.9, 115.6, 113.6, 112.6, 106.2. Anal. Calcd for C_14_H_8_ClN_3_: C, 66.28; H, 3.18; N, 16.56. Found: C, 65.94; H, 3.33; N, 16.72.

*Ethyl 8-chloro-7-cyanoimidazo[1,2-a]pyridine-2-carboxylate* (**10**). Method E. **1b** (400 mg, 1.14 mmol) was used as starting material. The reaction mixture was heated at 200 °C by microwave irradiation. M.p. = 229 °C. 63% yield. ^1^H-NMR (CDCl_3_) δ 8.35 (s, 1H, H-3), 8.20 (d, 1H, *J* = 7.0 Hz, H-5), 7.05 (d, 1H, *J* = 7.0 Hz, H-6), 4.50 (q, 2H, *J* = 7.5 Hz, CH_2_), 1.45 (t, 3H, *J* = 7.5 Hz, CH_3_). ^13^C-NMR (CDCl_3_) δ 161.9, 141.3, 139.8, 131.4, 125.3, 120.0, 114.4, 114.0, 109.3, 61.8, 14.3. Anal. Calcd for C_11_H_8_ClN_3_O_2_: C, 52.92; H, 3.23; N, 16.83. Found: C, 53.16; H, 3.48; N, 16.97.

*7-Chloro-2-phenylimidazo[1,2-a]pyridine-8-carbonitrile* (**11**). Method E. **1d** (400 mg, 1.13 mmol) was used as starting material. The reaction mixture was heated at 150 °C by microwave irradiation. M.p. = 197 °C. 69% yield. ^1^H-NMR (CDCl_3_) δ 8.22 (d, 1H, *J* = 7.2 Hz, H-5), 7.96 (m, 2H, Ph-2,6), 7.91 (s, 1H, H-3), 7.45 (m, 2H, Ph-3,5), 7.37 (m, 1H, Ph-4), 6.90 (d, 1H, *J* = 7.2 Hz, H-6). ^13^C-NMR (CDCl_3_) δ 148.4, 143.3, 137.3, 132.2, 129.0, 128.8, 126.4, 113.5, 112.6, 109.3, 102.0 (1 C not found). Anal. Calcd for C_14_H_8_ClN_3_: C, 66.28; H, 3.18; N, 16.56. Found: C, 66.34; H, 3.35; N, 16.51.

*Ethyl 7-chloro-8-cyanoimidazo[1,2-a]pyridine-2-carboxylate* (**12**). Method E. **1e** (400 mg, 1.14 mmol) was used as starting material. The reaction mixture was heated at 150 °C by microwave irradiation. M.p. = 227 °C. 46% yield. ^1^H-NMR (CDCl_3_) δ 8.31 (d, 1H, *J* = 7.2 Hz, H-5), 8.28 (s, 1H, H-3), 7.06 (d, 1H, *J* = 7.4 Hz, H-6), 4.48 (q, 2H, *J* = 7.1 Hz, CH_2_), 1.45 (t, 3H, *J* = 7.1 Hz, CH_3_). ^13^C-NMR (CDCl_3_) δ 162.1, 139.8, 139.2, 129.6, 118.4, 115.3, 111.7, 61.8, 14.3 (2C not found). Anal. Calcd for C_11_H_8_ClN_3_O_2_: C, 52.92; H, 3.23; N, 16.83. Found: C, 53.17; H, 3.32; N, 16.79.

#### 3.2.6. General Procedure for Buchwald-Hartwig Cross-Coupling Reaction

*N-(8-Chloro-2-phenylimidazo[1,2-a]pyridin-7-yl)aniline* (**13**). Method F. To a solution of **1a** (100 mg, 0.28 mmol) in DME (3 mL) under argon, were added successively rac-BINAP (53 mg, 0.085 mmol), t-BuONa (81 mg, 0.85 mmol), Pd(PPh_3_)_4_ (33 mg, 0.03 mmol), CuI (10 mg, 0.053 mmol) and aniline (77 µL, 0.85 mmol). The mixture was heated at 85 °C by microwave irradiation for 45 min. After cooling to room temperature, water (50 mL) was added and the solution was extracted with EtOAc (3 × 50 mL), dried with anhydrous MgSO_4_ and evaporated under reduced pressure. The crude residues were purified by flash chromatography (silica, cyclohexane/EtOAc (7:3)). M.p. = 170 °C. 48% yield. ^1^H-NMR (CDCl_3_) δ 7.93 (m, 2H, Ph-2,6), 7.82 (d, 1H, *J* = 7.4 Hz, H-5), 7.67 (s, 1H, H-3), 7.47–7.28 (m, 5H, Ph-3,4,5, Ph-NH-3,5), 7.15 (m, 3H, Ph-NH-2,4,6), 6.75 (d, 1H, *J* = 7.4 Hz, H-6), 6.42 (s, 1H, NH). ^13^C-NMR (CDCl_3_) δ 139.7, 138.2, 133.4, 129.7, 128.6, 128.1, 126.2, 124.5, 124.3, 121.9, 120.1, 108.3, 104.3 (2C not found). Anal. Calcd for C_19_H_14_ClN_3_: C, 71.36; H, 4.41; N, 13.14. Found: C, 71.55; H, 4.39; N, 13.26.

*Ethyl 8-chloro-7-(cyclohexylamino)imidazo[1,2-a]pyridine-2-carboxylate* (**14**). Method G. To a solution of **1b** (100 mg, 0.29 mmol) in dioxane (3 mL), were added Pd_2_dba_3_ (11 mg, 0.01 mmol), Xantphos (20 mg, 0.03 mmol), K_2_CO_3_ (591 mg, 4.29 mmol) and cyclohexylamine (0.42 mL, 3.71 mmol). The mixture was irradiated under microwaves at 150 °C during 1 h 15 min. After cooling to room temperature, EtOAc (50 mL) and water (50 mL) were added. The solution was extracted with EtOAc (3 × 50 mL), dried with anhydrous MgSO_4_ and evaporated under reduced pressure. The crude residues were purified by flash chromatography [silica, cyclohexane/EtOAc (7:3)]. M.p. = 176 °C. 40% yield. ^1^H-NMR (CDCl_3_) δ 7.98 (s, 1H, H-3), 7.90 (d, 1H, *J* = 7.5 Hz, H-5), 6.56 (d, 1H, *J* = 7.5 Hz, H-6), 4.57 (m, 1H, NH), 4.41 (q, 2H, *J* = 7.0 Hz, CH_2_), 3.37 (m, 1H, CyHex), 2.02 (m, 2H, CyHex), 1.80 (m, 2H, CyHex), 1.66 (m, 1H, CyHex), 1.32 (m, 9H, CH_3_, CyHex). ^13^C-NMR (CDCl_3_) δ 163.3, 144.7, 141.3, 136.6, 124.8, 116.5, 104.2, 99.6, 60.9, 51.8, 33.6, 25.4, 24.6, 14.4. Anal. Calcd for C_16_H_20_ClN_3_O_2_: C, 59.72; H, 6.26; N, 13.06. Found: C, 59.88; H, 6.23; N, 12.97.

*N-(7-Chloro-2-phenylimidazo[1,2-a]pyridin-8-yl)aniline* (**15**). Method F. **1d** (100 mg, 0.28 mmol) was used as starting material. M.p. = 167 °C. 51% yield. ^1^H-NMR (CDCl_3_) δ 7.92 (m, 2H, Ph-2,6), 7.82 (s, 1H, H-3), 7.76 (d, 1H, *J* = 7.2 Hz, H-5), 7.43 (m, 2H, Ph-3,5), 7.32 (m, 3H, Ph-4, Ph-NH-3,5), 7.00 (m, 4H, Ph-NH-2,6,4, NH), 6.79 (d, 1H, *J* = 7.2 Hz, H-6). ^13^C-NMR (CDCl_3_) δ 145.3, 142.2, 141.6, 133.2, 128.7, 128.6, 128.1, 127.3, 126.0, 121.9, 119.4, 119.2, 119.0, 115.7, 109.3. Anal. Calcd for C_16_H_20_ClN_3_O_2_: C, 59.72; H, 6.26; N, 13.06. Found: C, 59.88; H, 6.23; N, 12.97.

*Ethyl 7-chloro-8-(cyclohexylamino)imidazo[1,2-a]pyridine-2-carboxylate* (**16**). Method G. **1e** (100 mg, 0.29 mmol) was used as starting material. M.p. = 90 °C. 66% yield. ^1^H-NMR (CDCl_3_) δ 8.03 (s, 1H, H-3), 7.49 (d, 1H, *J* = 7.2 Hz, H-5), 6.77 (d, 1H, *J* = 7.2 Hz, H-6), 4.43 (q, 2H, *J* = 7.0 Hz, CH_2_), 2.05 (m, 2H, CyHex), 1.77 (m, 2H, CyHex), 1.63 (m, 2H, NH, CyHex), 1.28 (m, 9H, CH_3_, CyHex). ^13^C-NMR (CDCl_3_) δ 162.0, 157.0, 144.9, 133.7, 126.7, 118.5, 117.8, 114.7, 61.3, 53.2, 34.5, 25.6, 24.9, 14.3. Anal. Calcd for C_16_H_20_ClN_3_O_2_: C, 59.72; H, 6.26; N, 13.06. Found: C, 59.95; H, 6.32; N, 13.01.

#### 3.2.7. Substitution of the Chlorine Atom in Position 8 of Compounds **2–6**, **9–10**, **14**

*Ethyl 7-(4-fluorophenyl)-8-(p-tolyl)imidazo[1,2-a]pyridine-2-carboxylate* (**17**). Method H. To a solution of **3** (100 mg, 0.31 mmol) in a mixture of dioxane (0.5 mL) and ethanol (0.3 mL), were added p-tolylboronic acid (60 mg, 0.44 mmol), K_2_CO_3_ (88 mg, 0.63 mmol) and Pd(PPh_3_)_4_ (36 mg, 0.031 mmol). The mixture was heated at 150 °C by microwave irradiation for 15 min. After cooling to room temperature, water (50 mL) was added and the solution was extracted with CH_2_Cl_2_ (3 × 50 mL). The organic layer was dried with anhydrous MgSO_4_ and evaporated under reduced pressure. The crude residue was purified by column chromatography (silica gel, CH_2_Cl_2_). M.p. = 241 °C. 95% yield. ^1^H-NMR (CDCl_3_) δ 8.25 (s, 1H, H-3), 8.19 (d, 1H, *J* = 7.2 Hz, H-5), 7.22 (d, 2H, *J* = 7.9 Hz, CH_3_-Ph-2,6), 7.12 (dd, *J* = 8.7–5.4 Hz, 2H, F-Ph-2,6), 7.03 (d, 2H, *J* = 7.9 Hz, CH_3_-Ph-3,5), 6.92 (m, 3H, F-Ph-3,5, H-6), 4.39 (q, 2H, *J* = 7.2 Hz, CH_2_), 2.30 (s, 3H, CH_3_), 1.38 (t, 3H, *J* = 7.2 Hz, CH_3_). ^13^C-NMR (CDCl_3_) δ 163.1, 161.9, 145.3, 137.6, 137.2, 135.9, 135.2, 131.3, 131.0, 129.3, 128.7, 124.5, 117.3, 117.2, 115.2, 61.0, 21.2, 14.3. (1C not found) Anal. Calcd for C_23_H_19_FN_2_O_2_: C, 73.78; H, 5.11; N, 7.48. Found: C, 74.03; H, 5.08; N, 7.56. 

*Ethyl 7-(4-fluorophenyl)-8-(4-methoxyphenyl)imidazo[1,2-a]pyridine-2-carboxylate* (**18**). Method H. 4-Methoxyphenylboronic acid (96 mg, 0.63 mmol) was used. The crude residue was purified by column chromatography (silica gel, CH_2_Cl_2_). M.p. = 199 °C. 76% yield. ^1^H-NMR (CDCl_3_) δ 8.26 (s, 1H, H-3), 8.20 (d, 1H, *J* = 7.2 Hz, H-5), 7.28 (d, 2H, *J* = 8.7 Hz, CH_3_O-Ph-2,6), 7.12 (dd, 2H, *J* = 8.7–5.4 Hz, F-Ph-2,6), 6.96–6.91 (m, 3H, H-6, F-Ph-3,5), 6.78 (d, 2H, *J* = 8.7 Hz, CH_3_O-Ph-3,5), 4.40 (q, 2H, *J* = 7.2 Hz, CH_2_), 3.78 (s, 3H, CH_3_O), 1.39 (t, 3H, *J* = 7.2 Hz, CH_3_). ^13^C-NMR (CDCl_3_) δ 162.6, 161.9, 159.1, 144.9, 136.6, 136.3, 134.7, 132.3, 131.2, 128.3, 125.9, 124.9, 117.5, 117.4, 115.2, 113.3, 61.1, 55.2, 14.3. Anal. Calcd for C_23_H_19_FN_2_O_3_: C, 70.76; H, 4.91; N, 7.18. Found: C, 70.84; H, 5.11; N, 7.01. 

*7-(4-Fluorophenyl)-8-(p-tolyl)-2-phenylimidazo[1,2-a]pyridine* (**19**). Method I. To a solution of **2** (100 mg, 0.31 mmol) in a mixture of DME (2 mL) and water (1 mL), were added p-tolylboronic acid (84 mg, 0.62 mmol), Na_2_CO_3_ (66 mg, 0.62 mmol) and Pd(PPh_3_)_4_ (18 mg, 0.016 mmol, 5 mol%). The mixture was heated at 120 °C for 30 min by microwave irradiation. After cooling to room temperature, water (50 mL) was added and the solution was extracted with CH_2_Cl_2_ (3 × 50 mL). The organic layer was dried with anhydrous MgSO_4_ and evaporated under reduced pressure. The crude residue was purified by column chromatography (silica gel, CH_2_Cl_2_). M.p. = 222 °C. 90% yield. ^1^H-NMR (CDCl_3_) δ 8.11 (d, 1H, *J* = 6.9 Hz, H-5), 7.95 (m, 2H, Ph-2,6), 7.91 (s, 1H, H-3), 7.42–7.28 (m, 5H, CH_3_-Ph-2,6, Ph-3,4,5), 7.17–7.11 (m, 4H, F-Ph-2,6, CH_3_-Ph-3,5), 6.94 (t, 2H, *J* = 8.7 Hz, F-Ph-3,5), 6.85 (d, 1H, *J* = 6,9 Hz, H-6), 2.36 (s, 3H, CH_3_). ^13^C-NMR (CDCl_3_) δ 161.8, 146.3, 145.4, 137.3, 135.9, 134.6, 133.7, 131.7, 131.4, 131.3, 128.6, 128.5, 127.8, 126.3, 123.9, 115.7, 115.1, 108.3, 21.3 (1C not found). Anal. Calcd for C_26_H_19_FN_2_: C, 82.52; H, 5.06; N, 7.40. Found: C, 82.77; H, 5.39; N, 7.28.

*7-(4-Fluorophenyl)-2-methyl-8-(p-tolyl)imidazo[1,2-a]pyridine* (**20**). Method I. **4** (100 mg, 0.38 mmol), p-tolylboronic acid (104 mg, 0.77 mmol), Na_2_CO_3_ (82 mg, 0.77 mmol, 2 eq) and Pd(PPh_3_)_4_ (22 mg, 0.02 mmol, 5 mol%) were used. The mixture was heated for 60 min. The crude residue was purified by flash chromatography (silica, CH_2_Cl_2_/MeOH (99:1)). M.p. = 195 °C. 84% yield. ^1^H-NMR (CDCl_3_) δ 8.11 (d, 1H, *J* = 7.2 Hz, H-5), 7.45 (s, 1H, H-3), 7.18 (d, 2H, *J* = 8.1 Hz, CH_3_-Ph-2,6), 7.11–7.08 (m, 4H, F-Ph-2,6, CH_3_-Ph-3,5), 6.90 (t, 2H, *J* = 8.7 Hz, F-Ph-3,5), 6.82 (d, 1H, *J* = 7.2 Hz, H-6), 2.44 (s, 3H, CH_3_), 2.29 (s, 3H, CH_3_). ^13^C-NMR (CDCl_3_) δ 161.7, 144.6, 143.7, 137.1, 135.7, 134.3, 131.8, 131.3, 130.7, 128.7, 127.3, 123.7, 114.9, 114.9, 109.9, 21.2, 14.4. Anal. Calcd for C_21_H_17_FN_2_: C, 79.72; H, 5.42; N, 8.85. Found: C, 79.95; H, 5.48; N, 8.75.

*7-(4-Fluorophenyl)-8-(4-methoxyphenyl)-2-phenylimidazo[1,2-a]pyridine* (**21**). Method I. **2** (100 mg, 0.31 mmol), 4-methoxyphenylboronic acid (94 mg, 0.62 mmol) were used. The mixture was heated for 30 min. The crude residue was purified by column chromatography (silica gel, CH_2_Cl_2_). M.p. = 201 °C. 82% yield. ^1^H-NMR (CDCl_3_) δ 8.15 (d, 1H, *J* = 7.0 Hz, H-5), 7.99–7.89 (m, 3H, Ph-2,6, H-3), 7.43–7.27 (m, 5H, Ph-3,4,5, CH_3_O-Ph-2,6), 7.12 (dd, 2H, *J* = 9–5.4 Hz, F-Ph-2,6), 6.94 (t, 2H, *J* = 8.7 Hz, F-Ph-3,5), 6.89–6.78 (m, 3H, CH_3_O-Ph-3,5, H-6), 3.81 (s, 3H, CH_3_O). ^13^C-NMR (CDCl_3_) δ 161.9, 159.0, 146.0, 145.3, 135.9, 134.8, 133.5, 132.7, 131.4, 128.6, 127.9, 126.9, 126.3, 124.0, 115.8, 115.2, 113.4, 108.5, 55.2 (1 C not found). Anal. Calcd for C_26_H_19_FN_2_O: C, 79.17; H, 4.86; N, 7.10. Found: C, 79.35; H, 5.91; N, 7.24.

*7-(4-Fluorophenyl)-2-methyl-8-(pyridin-4-yl)imidazo[1,2-a]pyridine* (**22**). Method I. **4** (100 mg, 0.38 mmol), pyridin-4-ylboronic acid (76 mg, 0.54 mmol), Na_2_CO_3_ (82 mg, 0.77 mmol) and Pd(PPh_3_)_4_ (22 mg, 0.02 mmol, 5 mol%) were used. The mixture was heated for 60 min. The crude residue was purified by flash chromatography (silica, CH_2_Cl_2_/MeOH (99:1)). M.p. = 188 °C. 80% yield. ^1^H-NMR (CDCl_3_) δ 8.54 (d, 2H, *J* = 6.0 Hz, Pyr-2,6), 8.15 (d, 1H, *J* = 6.9 Hz, H-5), 7.46 (s, 1H, H-3), 7.31 (dd, 2H, *J* = 6.0 Hz, Pyr-3,5), 7.08 (dd, 2H, *J* = 8.7–5.4 Hz, F-Ph-2,6), 6.94 (t, 2H, *J* = 8.7 Hz, F-Ph-3,5), 6.87 (d, 1H, *J* = 6.9 Hz, H-6), 2.47 (s, 3H, CH_3_). ^13^C-NMR (CDCl_3_) δ 162.2, 148.9, 144.1, 143.2, 135.7, 134.4, 131.3, 126.2, 125.0, 124.0, 115.7, 115.5, 110.3, 14.2 (1C not found). Anal. Calcd for C_19_H_14_FN_3_: C, 75.23; H, 4.65; N, 13.85. Found: C, 75.52; H, 4.56; N, 13.98.

*8-(Fur-2-yl)-7-(p-tolylethynyl)-2-phenylimidazo[1,2-a]pyridine* (**23**). Method I. **5** (100 mg, 0.29 mmol), fur-2-ylboronic acid (65 mg, 0.58 mmol), Na_2_CO_3_ (62 mg, 0.58 mmol) and Pd(PPh_3_)_4_ (17 mg, 0.02 mmol, 5 mol%) were used. The mixture was heated for 75 min. The crude residue was purified by flash chromatography [silica, cyclohexane/EtOAc (7:3)]. M.p. > 300 °C. 19% yield. ^1^H-NMR (300 MHz, CDCl_3_) δ 8.08–7.96 (m, 4H, H-5, Ph-2,6, Fur-3), 7.91 (s, 1H, H-3) 7.74 (d, 1H, *J* = 1.7–0.9 Hz, Fur-5), 7.53–7.40 (m, 4H, Ph-3,5, CH_3_-Ph-2,6), 7.34 (m, 1H, Ph-4), 7.21 (d, 2H, *J* = 7.9 Hz, CH_3_-Ph-3,5), 6.98 (d, 1H, *J* = 6.8 Hz, H-6), 6.70 (dd, 1H, *J* = 3.4–1.7 Hz, Fur-4), 2.41 (s, 3H, CH_3_). ^13^C-NMR (CDCl_3_) δ 148.6, 145.8, 143.0, 139.0, 133.0, 131.5, 129.2, 128.7, 128.2, 126.2, 123.0, 120.5, 120.2, 117.2, 115.2, 114.5, 111.9, 109.4, 96.0, 88.5, 21.6 (1C not found). Anal. Calcd for C_26_H_18_N_2_O: C, 83.40; H, 4.85; N, 7.48. Found: C, 83.56; H, 4.91; N, 7.34.

*2-Phenyl-8-(thien-3-yl)imidazo[1,2-a]pyridine-7-carbonitrile* (**24**). Method I. **9** (100 mg, 0.40 mmol), thien-3-ylboronic acid (71 mg, 0.55 mmol), Na_2_CO_3_ (84 mg, 0.79 mmol) and Pd(PPh_3_)_4_ (23 mg, 0.02 mmol, 5 mol%) were used. The mixture was heated for 15 min. The crude residue was purified by flash chromatography (silica, petroleum ether/EtOAc (100:0) to (20:80)). M.p. = 193 °C. 71% yield. ^1^H-NMR (CDCl_3_) δ 8.48 (dd, 1H, *J* = 3.0–1.3 Hz, Th-2), 8.11 (d, 1H, *J* = 7.0 Hz, H-5), 8.05 (dd, 1H, *J* = 5.1–1.3 Hz, Th-4), 7.99 (m, 3H, H-3, Ph-2,6), 7.52 (dd, 1H, *J* = 5.1–3.0 Hz, Th-5), 7.45 (m, 2H, Ph-3,5), 7.37 (m, 1H, Ph-4), 7.01 (d, 1H, *J* = 7.0 Hz, H-6). ^13^C-NMR (CDCl_3_) δ 148.3, 142.7, 132.7, 132.5, 130.8, 129.7, 129.0, 128.8, 128.7, 126.3, 125.3, 124.0, 118.6, 114.3, 110.6, 103.2. Anal. Calcd for C_18_H_11_N_3_S: C, 71.74; H, 3.68; N, 13.94. Found: C, 71.62; H, 3.77; N, 14.05.

*2-Phenyl-8-(pyridin-4-yl)imidazo[1,2-a]pyridine-7-carbonitrile* (**25**). Method I. **9** (100 mg, 0.40 mmol), pyridin-4-ylboronic acid (78 mg, 0.55 mmol), Na_2_CO_3_ (84 mg, 0.79 mmol), Pd(PPh_3_)_4_ (23 mg, 0.02 mmol, 5 mol%) were used. The mixture was heated for 15 min. The crude residue was purified by flash chromatography [silica, cyclohexane/EtOAc (7:3)]. M.p. = 278 °C. 42% yield. ^1^H-NMR (CDCl_3_) δ 8.84 (d, 2H, *J* = 5.8 Hz, Pyr-2,6), 8.78 (d, 1H, *J* = 7.0 Hz, H-5), 8.74 (s, 1H, H-3), 7.96 (m, 2H, Ph-2,6), 7.84 (m, 2H, Pyr-3,5), 7.47 (m, 2H, Ph-3,5), 7.36 (m, 2H, H-6, Ph-4). ^13^C-NMR (CDCl_3_) δ 149.8, 147.5, 141.8, 140.7, 132.6, 132.4, 128.9, 128.6, 127.8, 126.0, 124.7, 117.4, 113.3, 112.5, 104.9. Anal. Calcd for C_19_H_12_N_4_: C, 77.01; H, 4.08; N, 18.91. Found: C, 77.12; H, 3.97; N, 18.89.

*Ethyl 7-(cyclohexylamino)-8-(p-tolyl)imidazo[1,2-a]pyridine-2-carboxylate* (**26**). Method H. **14** (100 mg, 0.31 mmol), p-tolylboronic acid (84.3 mg, 0.62 mmol) were used. The crude residue was purified by flash chromatography [silica, cyclohexane/EtOAc (7:3)]. M.p. = 169 °C. 27% yield. ^1^H-NMR (CDCl_3_) δ 8.04 (d, 1H, *J* = 7.5 Hz, H-5), 8.00 (s, 1H, H-3), 7.35 (d, 2H, *J* = 8.0 Hz, CH_3_-Ph-2,6), 7.25 (d, 2H, *J* = 8.0 Hz, CH_3_-Ph-3,5), 6.65 (d, 1H, *J* = 7.5 Hz, H-6), 4.34 (q, 2H, *J* = 7.0 Hz, CH_2_), 4.15 (s, 1H, NH), 3.32 (m, 1H, CyHex), 2.39 (s, 3H, CH_3_), 1.94 (m, 2H, CyHex), 1.64 (m, 3H, CyHex), 1.20 (m, 8H, CH_3_, CyHex). ^13^C-NMR (CDCl_3_) δ 163.4, 146.9, 142.5, 137.7, 135.8, 130.4, 129.9, 129.6, 125.6, 115.7, 108.4, 105.3, 60.7, 52.1, 33.6, 25.5, 24.7, 21.3, 14.3. Anal. Calcd for C_23_H_27_FN_3_O_2_: C, 73.18; H, 7.21; N, 11.13. Found: C, 73.39; H, 7.18; N, 11.09.

*Ethyl 8-(1H-indol-6-yl)-7-(p-tolylethynyl)imidazo[1,2-a]pyridine-2-carboxylate* (**27**). Method H. **6** (100 mg, 0.3 mmol), indol-6-ylboronic acid (96.6 mg, 0.6 mmol), K_2_CO_3_ (83 mg, 0.59 mmol) and Pd(PPh_3_)_4_ (34 mg, 0.03 mmol) were used. The crude residue was purified by flash chromatography [silica, cyclohexane/EtOAc (7:3)]. M.p. = 242 °C. 35% yield. ^1^H-NMR (CDCl_3_) δ 10.17 (bs, 1H, NH), 8.17–8.09 (m, 2H, H-5, H-3), 7.78 (s, 1H, Indol-7), 7.39 (d, 1H, *J* = 8.1 Hz, Indol-4*), 7.24 (d, 1H, *J* = 8.1 Hz, Indol-5*), 7.19–6.92 (m, 6H, H-6, CH_3_-Ph, Indol-2), 6.33 (s, 1H, Indol-3), 4.27 (q, 2H, *J* = 7.1 Hz, CH_2_), 2.27 (s, 3H, CH_3_), 1.14 (t, 3H, *J* = 7.1 Hz, CH_3_). ^13^C-NMR (CDCl_3_) δ 161.6, 143.7, 139.2, 135.4, 134.6, 133.5, 131.6, 129.1, 128.1, 126.2, 125.6, 124.5, 121.2, 119.2, 119.0, 118.6, 118.0, 114.0, 101.1, 97.0, 87.0, 61.5, 21.5, 14.0. (1 C not found) Anal. Calcd for C_27_H_21_N_3_O_2_: C, 77.31; H, 5.05; N, 10.02. Found: C, 77.47; H, 4.98; N, 10.13. 

*Ethyl 7-cyano-8-(pyridin-4-yl)imidazo[1,2-a]pyridine-2-carboxylate* (**28**). Method H. **10** (100 mg, 0.4 mmol), pyridin-4-ylboronic acid (80 mg, 0.56 mmol), K_2_CO_3_ (112 mg, 0.8 mmol), Pd(PPh_3_)_4_ (46 mg, 0.04 mmol) were used. The crude residue was purified by flash chromatography [silica, cyclohexane/EtOAc (100:0) to (10:90)]. M.p. = 185 °C. 31% yield. ^1^H-NMR (CDCl_3_) δ 8.86 (d, 2H, J = 6.0 Hz, Pyr-2,6), 8.40 (s, 1H, H-3), 8.35 (d, 1H, *J* = 7.0 Hz, H-5), 7.91 (d, 2H, *J* = 6.0 Hz, Pyr-3,5), 7.19 (d, 1H, *J* = 7.0 Hz, H-6), 4.45 (q, 2H, *J* = 7.1 Hz, CH_2_), 1.41 (t, 3H, *J* = 7.1 Hz, CH_3_). ^13^C-NMR (CDCl_3_) δ 162.2, 149.9, 142.2, 139.9, 126.8, 124.6, 119.3, 116.4, 115.0, 107.5, 61.7, 14.3. (2C not found) Anal. Calcd for C_16_H_12_N_4_O_2_: C, 65.75; H, 4.14; N, 19.17. Found: C, 65.92; H, 4.28; N, 19.04.

*Ethyl 7-cyano-8-(fur-2-yl)imidazo[1,2-a]pyridine-2-carboxylate* (**29**). Method H. **10** (100 mg, 0.4 mmol), fur-2-ylboronic acid (63 mg, 0.56 mmol), K_2_CO_3_ (112 mg, 0.8 mmol), Pd(PPh_3_)_4_ (46 mg, 0.04 mmol) were used. The crude residue was purified by flash chromatography [silica, cyclohexane/EtOAc (7:3)]. M.p. = 185 °C. 34% yield. ^1^H-NMR (CDCl_3_) δ 8.29–8.22 (m, 2H, H-3, Fur-3), 8.07 (d, 1H, *J* = 7.2 Hz, H-5), 7.78 (dd, 1H, *J* = 1.7–0.8 Hz, Fur-5), 7.08 (d, 1H, *J* = 7.2 Hz, H-6), 6.71 (dd, 1H, *J* = 3.6–1.7 Hz, Fur-4), 4.48 (q, 2H, *J* = 7.2 Hz, CH_2_), 1.46 (t, 3H, *J* = 7.2 Hz, CH_3_). ^13^C-NMR (CDCl_3_) δ 162.4, 148.8, 145.3, 140.1, 138.5, 123.7, 122.2, 119.3, 118.9, 117.9, 116.2, 113.0, 61.5, 14.3. (1 C not found) Anal. Calcd for C_15_H_11_N_3_O_3_: C, 64.05; H, 3.94; N, 14.94. Found: C, 63.92; H, 4.08; N, 15.02.

*Ethyl 7-(cyclohexylamino)-8-(pyridin-4-yl)imidazo[1,2-a]pyridine-2-carboxylate* (**30**). Method H. **14** (100 mg, 0.31 mmol), pyridin-4-ylboronic acid (76.2 mg, 0.62 mmol) were used. The crude residue was purified by flash chromatography [silica, cyclohexane/EtOAc (6:4)]. M.p. = 182 °C. 28% yield. ^1^H-NMR (CDCl_3_) δ 8.72 (d, 2H, *J* = 5.1 Hz, Pyr-2,6), 7.98 (m, 2H, H-5, H-3), 7.57 (d, 2H, *J* = 5.1 Hz, Pyr-3,5), 6.65 (d, 1H, *J* = 7.7 Hz, H-6), 4.35 (q, 2H, *J* = 7.0 Hz, CH_2_), 4.26 (m, 1H, NH), 3.35 (m, 1H, CyHex), 1.91 (m, 2H, CyHex), 1.66 (m, 3H, CyHex), 1.40–1.05 (m, 8H, CH_3_, CyHex). ^13^C-NMR (CDCl_3_) δ 163.4, 150.6, 149.9, 146.0, 142.3, 136.4, 126.6, 125.8, 121.4, 115.9, 105.0, 60.9, 52.0, 33.5, 25.4, 24.7, 14.3. Anal. Calcd for C_21_H_24_N_4_O_2_: C, 69.21; H, 6.64; N, 15.37. Found: C, 69.38; H, 6.71; N, 15.24.

*7-(4-Methoxyphenyl)-2-phenyl-8-(p-tolylethynyl)imidazo[1,2-a]pyridine* (**31**). Method I. **7** (100 mg, 0.29 mmol), 4-methoxyphenylboronic acid (88 mg, 0.58 mmol), Na_2_CO_3_ (62 mg, 0.58 mmol), Pd(PPh_3_)_4_ (17 mg, 0.014 mmol, 5 mol%) were used. The mixture was heated for 30 min. The crude residue was purified by flash chromatography [silica, cyclohexane/EtOAc (7:3)]. M.p. = 84 °C. 56% yield. ^1^H-NMR (CDCl_3_) δ 8.12–8.01 (m, 3H, H-5, Ph-2,6), 7.87 (s, 1H, H-3), 7.75 (m, 2H, CH_3_O-Ph-2,6), 7.46 (m, 4H, Ph-3,5, CH_3_-Ph-2,6), 7.34 (m, 1H, Ph-4), 7.16 (d, 2H, *J* = 7.7 Hz, CH_3_-Ph-3,5), 7.03 (m, 2H, CH_3_O-Ph-3,5), 6.89 (d, 1H, *J* = 7.0 Hz, H-6), 3.89 (s, 3H, CH_3_O), 2.38 (s, 3H, CH_3_). ^13^C-NMR (CDCl_3_) δ 159.7, 146.3, 145.8, 140.2, 138.7, 133.5, 131.7, 131.0, 130.5, 129.0, 128.6, 128.0, 126.3, 124.4, 120.2, 114.5, 113.6, 109.6, 108.4, 98.6, 84.3, 55.4, 21.6. Anal. Calcd for C_29_H_22_N_2_O: C, 84.03; H, 5.35; N, 6.76. Found: C, 84.13; H, 5.34; N, 6.81.

*Ethyl 7-(4-methoxyphenyl)-8-(p-tolylethynyl)imidazo[1,2-a]pyridine-2-carboxylate* (**32**). Method H. **8** (100 mg, 0.30 mmol), 4-methoxyphenylboronic acid (65 mg, 0.42 mmol) were used. The crude residue was purified by flash chromatography [silica, cyclohexane/EtOAc (7:3)]. M.p. = 240 °C. 69% yield. ^1^H-NMR (CDCl_3_) δ 8.22 (s, 1H, H-3), 8.13 (d, 1H, *J* = 7.2 Hz, H-5), 7.75 (d, 2H, *J* = 9 Hz, CH_3_O-Ph-2,6), 7.41 (d, 2H, *J* = 8.1 Hz, CH_3_-Ph-2,6), 7.12 (d, 2H, *J* = 8.1 Hz, CH_3_O-Ph-3,5), 7.08–6.98 (m, 3H, H-6, CH_3_-Ph-3,5), 4.47 (q, 2H, *J* = 7.2 Hz, CH_2_), 3.89 (s, 3H, CH_3_O), 2.35 (s, 3H, CH_3_), 1.46 (t, 3H, *J* = 7.2 Hz, CH_3_). ^13^C-NMR (CDCl_3_) δ 163.0, 160.0, 145.5, 141.8, 138.9, 137.1, 131.8, 130.6, 130.4, 129.0, 124.8, 119.9, 117.6, 116.3, 113.7, 110.9, 99.6, 83.6, 61.3, 55.4, 21.6, 14.4. Anal. Calcd for C_26_H_22_N_2_O_3_: C, 76.08; H, 5.40; N, 6.82. Found: C, 76.24; H, 5.29; N, 6.64.

*7-(4-Methoxyphenyl)-2-phenylimidazo[1,2-a]pyridine-8-carbonitrile* (**33**). Method I. **11** (100 mg, 0.40 mmol), 4-methoxyphenylboronic acid (84 mg, 0.55 mmol), Na_2_CO_3_ (84 mg, 0.79 mmol) and Pd(PPh_3_)_4_ (23 mg, 0.02 mmol, 5 mol%) were used. The mixture was heated for 15 min. The crude residue was purified by flash chromatography [silica, cyclohexane/EtOAc (7:3)]. M.p. = 185 °C. 61% yield. ^1^H-NMR (CDCl_3_) δ 8.26 (d, 1H, *J* = 7.0 Hz, H-5), 8.00 (m, 2H, Ph-2,6), 7.88 (s, 1H, H-3), 7.62 (d, 2H, *J* = 9.0 Hz, CH_3_O-Ph-2,6), 7.44 (m, 2H, Ph-3,5), 7.35 (m, 1H, Ph-4), 7.02 (d, 2H, *J* = 9.0 Hz, CH_3_O-Ph-3,5), 6.90 (d, 1H, *J* = 7.0 Hz, H-6), 3.86 (s, 3 H, CH_3_O). ^13^C-NMR (CDCl_3_) δ 160.8, 147.5, 144.6, 144.1, 132.7, 130.0, 128.7, 128.5, 128.4, 128.3, 126.3, 115.5, 114.4, 113.5, 108.8, 98.1, 55.4. Anal. Calcd for C_21_H_15_N_3_O: C, 77.52; H, 4.65; N, 12.91. Found: C, 77.68; H, 4.53; N, 12.94.

*7-(4-Methylphenyl)-N,2-diphenylimidazo[1,2-a]pyridin-8-amine* (**34**). Method I. **15** (100 mg, 0.53 mmol), p-tolylboronic acid (362 mg, 2.66 mmol), Na_2_CO_3_ (112.4 mg, 1.06 mmol) and Pd(PPh_3_)_4_ (30.6 mg, 0.03 mmol, 5 mol%) were used. The mixture was heated for 30 min. The crude residue was purified by flash chromatography [silica, cyclohexane/EtOAc (70:30)] to give a white solid. M.p. = 237 °C. 44% yield. ^1^H-NMR (CDCl_3_) δ 7.98 (m, 2H, Ph-3,5), 7.87 (m, 2H, H-3, H-5), 7.45 (m, 2H, Ph-2,6), 7.34 (m, 3H, Ph-4, CH_3_-Ph-2,6), 7.00 (m, 4H, CH_3_-Ph-3,5, Ph-NH-3,5), 6.88 (d, 1H, *J* = 7.0 Hz, H-6), 6.72 (m, 3H, Ph-NH-2,4,6), 2.27 (s, 3H, CH_3_). ^13^C-NMR (CDCl_3_) δ 141.8, 136.9, 135.4, 131.3, 130.0, 129.6, 128.9, 128.7, 128.5, 128.1, 127.9, 126.1, 120.7, 118.6, 118.2, 116.8, 108.8, 21.1 (2C not found). Anal. Calcd for C_26_H_21_N_3_: C, 83.17; H, 5.64; N, 11.19. Found: C, 83.34; H, 5.77; N, 11.18.

#### 3.2.8. General Procedures for Double-Coupling Approach

*3-[8-(4-Methoxyphenyl)-2-phenylimidazo[1,2-a]pyridin-7-yl]aniline* (**35**). Method J. To a solution of **1a** (100 mg, 0.28 mmol) in DME (2 mL) and water (1 mL), were added 3-aminophenylboronic acid (39 mg, 0.28 mmol), Na_2_CO_3_ (90 mg, 0.85 mmol) and Pd(PPh_3_)_4_ (16 mg, 0.014 mmol). After irradiation under microwaves for 30 min at 95 °C, 4-methoxyphenylboronic acid (60 mg, 0.40 mmol) and Pd(PPh_3_)_4_ (16 mg, 0.014 mmol) were added. The mixture was irradiated under microwaves during 30 min at 120 °C. After cooling to room temperature, CH_2_Cl_2_ (50 mL) and water (50 mL) were added and the solution was extracted with CH_2_Cl_2_ (3 × 50 mL). The combined organic layer was dried with anhydrous MgSO_4_ and evaporated under reduced pressure. The crude residue was purified by flash chromatography (alumina, CH_2_Cl_2_) to give a yellow solid. M.p. = 230 °C. 44% yield. ^1^H-NMR (CDCl_3_) δ 8.10 (d, 1H, *J* = 7.0 Hz, H-5), 7.94 (m, 3H, H-3, Ph-3,5), 7.42 (m, 4H, Ph-2,6, CH_3_O-Ph-2,6), 7.31 (m, 1H, Ph-4), 7.02 (t, 1H, *J* = 7.7 Hz, H_2_N-Ph-5), 6.86 (m, 3H, H-6, CH_3_O-Ph-3,5), 6.55 (m, 3H, H_2_N-Ph-4,6,2), 3.81 (s, 3H, CH_3_O), 3.23 (bs, 2H, NH_2_). ^13^C-NMR (CDCl_3_) δ 158.8, 146.1, 146.0, 145.5, 141.0, 135.8, 133.7, 132.6, 130.6, 128.9, 128.5, 127.8, 127.3, 126.2, 123.6, 120.3, 116.4, 115.9, 113.8, 113.2, 108.2, 55.1. Anal. Calcd for C_26_H_21_N_3_O: C, 79.77; H, 5.41; N, 10.73. Found: C, 79.92; H, 5.38; N, 10.63.

*2-Phenyl-8-(pyridin-4-yl)-7-(thien-3-yl)imidazo[1,2-a]pyridine* (**36**). Method J. Thien-3-ylboronic acid (36 mg, 0.28 mmol, 1 eq) and pyridin-4-ylboronic acid (56 mg, 0.40 mmol, 1.4 eq) were used. The crude residue was purified by flash chromatography [silica, cyclohexane/EtOAc (99:1)] to give a white solid. M.p. = 273 °C. 43% yield. ^1^H-NMR (CDCl_3_) δ 8.62 (dd, 2 H, *J* = 4.5–1.5 Hz, Pyr-2,6), 8.16 (d, 1H, *J* = 7.0 Hz, H-5), 7.92 (m, 3 H, H-3, Ph-3,5), 7.49 (dd, 2 H, *J* = 4.5–1.5 Hz, Pyr-3,5), 7.40 (m, 2 H, Ph-2,6), 7.32 (m, 1 H, Ph-4), 7.21 (dd, 1H, *J* = 5.0–2.9 Hz, Th-5), 7.09 (dd, 1H, *J* = 2.9–1.4 Hz, Th-2), 6.97 (d, 1H, *J* = 7.0 Hz, H-6), 6.76 (dd, 1H, *J* = 5.0–1.4 Hz, Th-4). ^13^C-NMR (CDCl_3_) δ 149.0, 146.9, 144.6, 144.3, 139.1, 133.5, 130.9, 128.6, 128.5, 128.1, 126.2, 125.8, 125.0, 124.6, 124.5, 115.2, 108.5 (1C not found). Anal. Calcd for C_22_H_15_N_3_S: C, 74.76; H, 4.28; N, 11.89. Found: C, 74.89; H, 4.21; N, 11.78.

*8-(4-Fluorophenyl)-2-phenyl-7-(p-tolyl)imidaz*o*[1,2-a]pyridine* (**37**). Method K. To a solution of **1d** (100 mg, 0.28 mmol) in DME (2 mL) and water (1 mL), were added 4-fluorophenylboronic acid (79 mg, 0.56 mmol), Na_2_CO_3_ (90 mg, 0.85 mmol) and Pd(PPh_3_)_4_ (16 mg, 0.014 mmol). After irradiation under microwaves for 1 h 30 min at 95 °C, *p*-tolylboronic acid (77 mg, 0.56 mmol) and Pd(PPh_3_)_4_ (16 mg, 0.014 mmol) were added. The mixture was irradiated under microwaves for 30 min at 120 °C. After cooling to room temperature, CH_2_Cl_2_ (50 mL) and water (50 mL) were added and the solution was extracted with CH_2_Cl_2_ (3 × 50 mL). The organic layer was dried with anhydrous MgSO_4_ and evaporated under reduced pressure. The crude residue was purified by flash chromatography [silica, cyclohexane/EtOAc (99:1)] to give a white solid. M.p. = 215 °C. 47% yield. ^1^H-NMR (CDCl_3_) δ 8.14 (d, 1H, *J* = 7.0 Hz, H-5), 7.98–7.90 (m, 3H, H-3, Ph-3,5), 7.50–7.37 (m, 4H, Ph-2,6, F-Ph-3,5), 7.32 (m, 1H, Ph-4), 7.11–6.95 (m, 6H, F-Ph-2,6, CH_3_-Ph-2,3,5,6), 6.92 (d, 1H, *J* = 7.0 Hz, H-6), 2.35 (s, 3H, CH_3_). ^13^C-NMR (CDCl_3_) δ 162.1, 146.3, 145.4, 136.9, 136.5, 133.7, 133.2, 131.0, 129.8, 129.6, 128.9, 128.5, 127.9, 126.2, 124.1, 115.9, 115.1, 114.8, 108.3, 21.1. 

*2-Phenyl-7-(p-tolylethynyl)imidazo[1,2-a]pyridine-8-carbonitrile* (**38**). Method L. To a solution of **1d** (100 mg, 0.28 mmol) in DMF (3 mL), was added CuCN (31 mg, 0.34 mmol). The mixture was irradiated under microwaves at 90 °C during 4 h. Then, 4-ethynyltoluene (47 µL, 0.37 mmol), Et_3_N (198 µL, 1.41 mmol), Pd(PPh_3_)_4_ (33 mg, 0.03 mmol), PCy_3_HBF_4_ (31 mg, 0.08 mmol) and CuI (11 mg, 0.056 mmol) were added. The mixture was irradiated under microwaves at 130 °C during 1 h. After cooling to room temperature, a solution of NH_4_OH 10% (50 mL) and EtOAc (50 mL) were added. The organic layer was washed with the solution of NH_4_OH (3 × 50 mL), dried with anhydrous MgSO_4_ and evaporated under reduced pressure. The crude residues were purified by flash chromatography [silica, cyclohexane/EtOAc (7:3)]. M.p. = 214 °C. 32% yield. ^1^H-NMR (CDCl_3_) δ 8.22 (d, *J* = 7.0 Hz, 1H, H-5), 8.02 (m, 2H, Ph-2,6), 7.94 (s, 1H, H-3), 7.55 (m, 2H, CH_3_-Ph-2,6), 7.46 (m, 2H, Ph-3,5), 7.38 (m, 1H, Ph-4), 7.21 (d, 2H, *J* = 7.9 Hz, CH_3_-Ph-3,5), 6.95 (d, 1H, *J* = 7.0 Hz, H-6), 2.41 (s, 3H, CH_3_). ^13^C-NMR (CDCl_3_) δ 148.5, 140.4, 132.4, 132.2, 129.3, 128.8, 128.8, 128.7, 128.0, 127.7, 126.5, 125.2, 118.3, 114.4, 114.2, 109.7, 100.5, 84.8, 21.7. Anal. Calcd for C_23_H_15_N_3_: C, 82.86; H, 4.54; N, 12.60. Found: C, 82.97; H, 4.56; N, 12.68.

*7-[4-(4-Fluorophenyl)piperazin-1-yl]-2-phenylimidazo[1,2-a]pyridine-8-carbonitrile* (**39**). Method M. To a solution of **1d** (100 mg, 0.28 mmol) in DMF (3 mL), CuCN (31 mg, 0.34 mmol) was added. The mixture was stirred at 90 °C during 4 h under microwave irradiation. Then DIPEA (0.1 mL, 0.57 mmol) and 4-fluorophenylpiperidine (98 mg, 0.57 mmol) were added. The mixture was heated at 130 °C during 1 h under microwaves irradiation. After cooling to room temperature, EtOAc (50 mL) and a solution of NH_4_OH 10% (50 mL) were added. The solution was extracted with EtOAc (3 × 50 mL), dried with anhydrous MgSO_4_ and evaporated under reduced pressure. The crude residue was purified by flash chromatography [silica, cyclohexane/EtOAc (7:3)]. M.p. = 224 °C. 67% yield. ^1^H-NMR (CDCl_3_) δ 8.06 (d, 1H, *J* = 7.5 Hz, H-5), 7.97 (m, 2H, Ph-2,6), 7.71 (s, 1H, H-3), 7.42 (m, 2H, Ph-3,5), 7.33 (m, 1H, Ph-4), 7.06–6.89 (m, 4H, F-Ph), 6.55 (d, 1H, *J* = 7.5 Hz, H-6), 3.79–3.69 (m, 4H, pip), 3.36–3.27 (m, 4H, pip). ^13^C-NMR (CDCl_3_) δ 157.7, 154.0, 147.2, 146.6, 145.4, 133.0, 129.1, 128.7, 128.2, 126.2, 118.5, 116.0, 115.8, 107.6, 105.3, 85.3, 50.5, 50.2. Anal. Calcd for C_24_H_20_FN_5_: C, 72.53; H, 5.07; N, 17.62. Found: C, 72.49; H, 5.01; N, 18.73.

*Ethyl 8-cyano-7-(p-tolylethynyl)imidazo[1,2-a]pyridine-2-carboxylate* (**40**). Method L. **1e** (100 mg, 0.29 mmol) was used as starting material. M.p. = 93 °C. 43% yield. ^1^H-NMR (CDCl_3_) δ 8.22 (s, 1H, H-3), 8.12 (d, 1H, *J* = 7.2 Hz, H-5), 7.54 (d, 2H, *J* = 8.0 Hz, CH_3_-Ph-2,6), 7.17 (d, 2H, *J* = 8.0 Hz, CH_3_-Ph-3,5), 6.96 (d, 1H, *J* = 7.2 Hz, H-6), 4.44 (q, 2H, *J* = 7.2 Hz, CH_2_), 2.38 (s, 3H, CH_3_), 1.42 (t, 3H, *J* = 7.2 Hz, CH_3_). ^13^C-NMR (CDCl_3_) δ 162.6, 144.6, 139.6, 137.3, 135.3, 132.0, 129.0, 125.3, 119.1, 118.1, 116.0, 113.7, 102.9, 80.8, 61.4, 21.6, 14.3 (1 C not found). Anal. Calcd for C_20_H_15_N_3_O_2_: C, 72.94; H, 4.59; N, 12.76. Found: C, 73.27; H, 4.68; N, 12.81.

*Ethyl 8-cyano-7-[4-(4-fluorophenyl)piperazin-1-yl]imidazo[1,2-a]pyridine-2-carboxylate* (**41**). Method M. **1e** (100 mg, 0.29 mmol) was used as starting material. M.p. = 193 °C. 54% yield. ^1^H-NMR (CDCl_3_) δ 8.20–7.91 (m, 2H, H-3, H-5), 7.07–6.88 (m, 4H, F-Ph), 6.67 (d, 1H, *J* = 7.0 Hz, H-6), 4.43 (q, 2H, *J* = 7.0 Hz, CH_2_), 3.83 (bs, 4H, pip), 3.33 (bs, 4H, H-pip), 1.42 (t, 3H, *J* = 7.0 Hz, CH_3_). ^13^C-NMR (CDCl_3_) δ 162.8, 157.9, 154.4, 145.3, 137.5, 132.5, 129.5, 123.6, 118.7, 117.2, 115.9, 107.4, 84.5, 61.3, 50.7, 49.9, 14.3. Anal. Calcd for C_21_H_20_FN_5_O_2_: C, 64.11; H, 5.12; N, 17.80. Found: C, 64.49; H, 5.08; N, 17.94.

## 4. Conclusions

The reactivity of the 7-chloro-8-iodo- and 8-chloro-7-iodoimidazo[1,2-*a*]pyridines **1a**–**e** variously substituted on the 2 position, towards Suzuki-Miyaura, Sonogashira, and Buchwald-Hartwig cross-coupling reactions as well as cyanation was evaluated. Various methodologies are proposed to introduce aryl, heteroaryl, alkyne, amine or cyano groups in the two positions depending on the nature of the substituent present in position 2. The sensitivity of the 2-ester group obliged us to adapt the reaction conditions, notably when a base was required. Moreover, the presence of the 2-ester seemed to lower the reactivity of the 8 position towards the Sonogashira and the cyanation reactions. 

In both series, the substitution was totally regioselective due to the differences in reactivity between the iodine and chlorine atoms. Nevertheless, the difficulty in our cases was to substitute the chlorine atom in the second step. Until now, only hetero(aryl) groups could be introduced though Suzuki-Miyaura cross-coupling. We overcame this problem evaluating the both regioisomers in parallel. 

The double coupling approach was also studied allowing the one pot Suzuki/Suzuki, cyanation/Sonogashira and cyanation/Buchwald reactions leading to polyfunctionalized imidazo[1,2-*a*]pyridines. New heterogeneous double coupling approaches are under investigations.
